# Experimental Evidence for the Solid-State Nitrite-Ligand Photoisomerization Mechanism in Nickel(II) Square-Planar Complexes

**DOI:** 10.1021/acsomega.5c08267

**Published:** 2025-10-26

**Authors:** Krystyna A. Deresz, Artem Mikhailov, Joanna Jankowska, Lorenzo Donà, Bartolomeo Civalleri, Adam Krówczyński, Radosław Kamiński, Dominik Schaniel, Katarzyna N. Jarzembska

**Affiliations:** † University of Warsaw, Faculty of Chemistry, Żwirki i Wigury 101, 02-089 Warsaw, Poland; ‡ Université de Lorraine, CNRS, CRM2, 54500 Nancy, France; § Dipartimento di Chimica, Università di Torino, via Giuria 5, 10125 Torino, Italy

## Abstract

Two square-planar nitrite nickel­(II) photoswitches were designed and synthesized, referred to as **Ni-4d** and the related oxime **Ni-4d′**. In the ground-state single crystals of both compounds, the nitro form (Ni–N­(O)_2_) is the dominant one. For the two systems, it was possible to generate and detect both *endo*-nitrito and *exo*-nitrito linkage isomers using 530 nm light-emitting diode (LED) light at 100 K. Over time, the *endo*-nitrito form takes over, and almost 100% conversion can be achieved, as confirmed by photocrystallographic and infrared (IR) spectroscopic experiments. The stability of this isomer is similar for both systems, which is confirmed by the experimentally determined decay temperature (*T*_d_) values. Furthermore, when 660 nm LED light is applied at 90 K, the *exo*-nitrito form can be generated up to about 25% population with no admixture of the *endo*-nitrito isomer for **Ni-4d**. Based on the kinetic parameters, the *exo*-nitrito isomer is slightly less stable for **Ni-4d′**. In this work, we report for the first time the experimental evidence of the earlier theoretically predicted nitro-to-*endo*-nitrito mechanism via the *exo*-nitrito form for square-planar nickel­(II) nitrite coordination compounds in the solid state.

## Graphical abstract

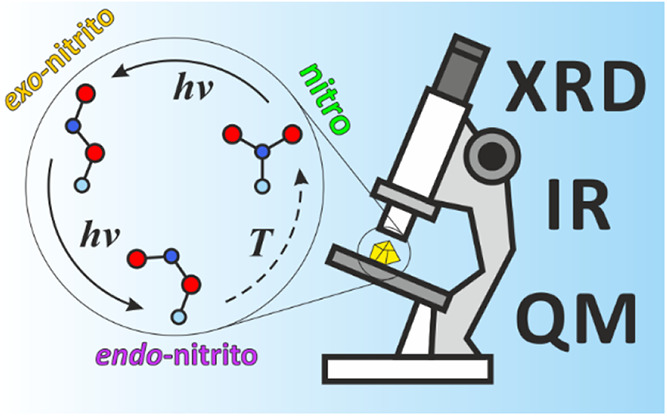

## 1 Introduction

The term photoswitchable compounds refers to materials that undergo various types of transformations under light irradiation, such as cyclization, *cis*-*trans* isomerization, or linkage isomerization.^1^ Thanks to the ability to transform from one form to another upon exposure to light, photoswitches may find wide applications in materials science,^2^ optoelectronics,^3^ or medicine.^4^ Transition-metal complexes containing ambidentate ligands, e.g., SO_2_, NO, and NO_2_,^5–9^ constitute an interesting and readily modifiable group of molecular photoswitches. The ambidentate ligands are key fragments in such molecules, as they may bind to a metal center in more than one way, depending on the conditions. For instance, the nitrite group may adopt four different binding modes in mononuclear complexes (Scheme 1): nitro-(η^1^-N­(O)_2_) (further referred to as nitro), *exo*-nitrito-(η^1^-ONO) (further referred to as *exo*-nitrito), *endo*-nitrito-(η^1^-ONO) (further referred to as *endo*-nitrito), and κ-nitrito-(η^2^-O_2_N),^10^ which, under some circumstances, may be interchanged.

**Scheme 1:**
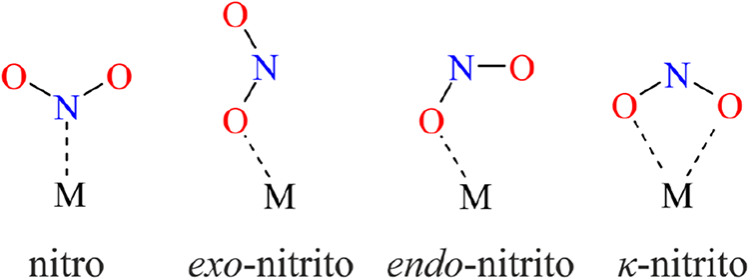
Selected Possible Coordination Modes of the Nitrite Group in Mononuclear Metal Complexes

Understanding of the influence of supporting ligands, metal centers, or crystal packing on the photoswitchable properties, as well as the isomerization reaction mechanism itself, is crucial in the context of designing new efficient photoswitches. To the best of our knowledge, to date, there is no direct experimental confirmation of the nitro-to-*endo*-nitrito reaction mechanism in square-planar nickel complexes. However, quite recently, Warren et al. proposed a mechanism according to which the nitro isomer transforms to the *exo*-nitrito form *first*, and subsequently converts to the final photoproduct, the *endo*-nitrito isomer.^11^ More recently, a possible isomerization reaction pathway for square-planar complexes was discussed in two other articles. The isomerization reaction mechanism fully consistent with the one described above was reported by us^12^ for crystals of the nickel nitro complex with the 1-phenyl-2-hydroxyimino-3-[(2′-dimethylamino)­ethyl]­imino-1-propanone moiety as an (*N*,*N*,*O*)-donor supporting ligand (**Ni-1d′**). The computationally evaluated mechanism (nitro → *exo*-nitrito → *endo*-nitrito) sheds light on the experimental observations. In contrast, Mikhailov et al.^13^ presented two possible reaction pathways based on the experiments performed for a series of complex salts of the [Pd­(NH_3_)_4_] [Pd­(NH_3_)_3_NO_2_] [M­(Ox)_3_]·*y* H_2_O type (M = Cr, Rh, Co; Ox = oxalate). In the first considered mechanism, the *endo*-nitrito isomer served as an intermediate product, while in the other potential pathway, the transformation went through the *exo*-nitrito isomer, similarly as suggested by the above-discussed papers.

Hence, in this paper, two related photoswitchable square-planar Ni^II^ nitrite complexes, namely **Ni-4d** and **Ni-4d′**, are introduced (Scheme 2). These compounds were designed based on the nickel nitro complexes with (*N*,*N*,*O*)-donor supporting ligands previously studied by some of us.^12,14,15^ The two compounds were crystallized and completely characterized, both experimentally and theoretically, in order to shed more light on the nitro-to-*endo*-nitrito photoreaction mechanism.

**Scheme 2:**
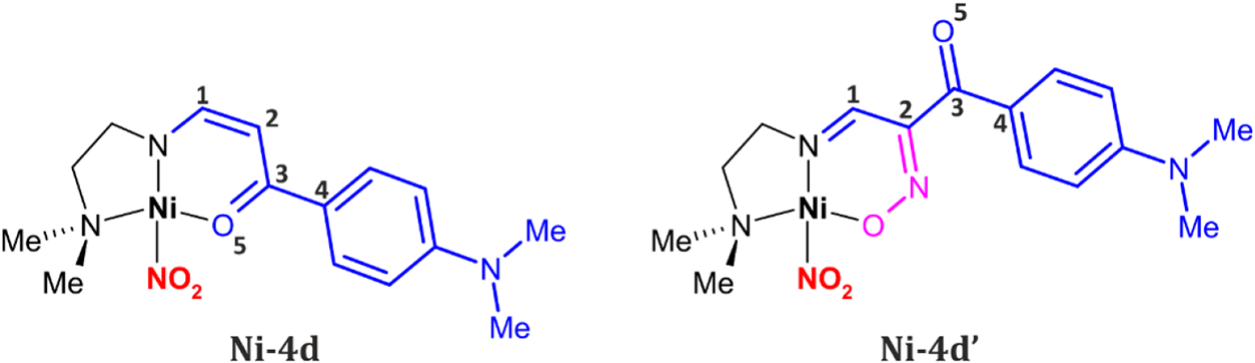
Schematic Representation of the Studied Systems: **Ni-4d** and **Ni-4d′** ^a^

a Corresponding molecular fragments are colored in blue, oxime fragment is shown in magenta; note the numbering of atoms underscores the difference between the two compounds.

## 2 Experimental Section

### 2.1 Synthesis

The compounds **Ni-4d** and **Ni-4d′** were synthesized in the same manner as in literature procedures^12,14^ using the following compounds to create a chelating ligand: 4-dimethyloaminoacetophenone, ethyl formate, and *N*,*N*-dimethyloethylenediamine. It is worth stressing that the **Ni-4d** compound is obtained when using a deficiency of LiNO_2_ in the reaction, and when an excess of LiNO_2_ is used, **Ni-4d′** is derived. The reaction steps are schematically presented in Figure S1 (Supporting Information). The yields of the reaction for **Ni-4d** and **Ni-4d′** were 55 and 49%, respectively.

### 2.2 X-ray Diffraction

All X-ray diffraction experiments (including the preliminary ones) were carried out on a Rigaku Oxford Diffraction SuperNova single-crystal diffractometer equipped with an Atlas CCD detector, a copper microfocus X-ray source (Cu–K_α_ radiation, λ = 1.54184 Å) coupled with the multilayer mirror optics, a low-temperature nitrogen gas flow Oxford Cryosystems device, and our homemade light-delivery device,^16^ which allows *in situ* photocrystallographic experiments. Single crystals were irradiated with 530 and 660 nm LED light (LED = light-emitting diode). The optimal data-collection strategy took into account the mounted light-delivery device and was automatically prepared by using the native diffractometer software. In the case of photocrystallographic experiments, the same strategy was used for all data collected for a given crystal (only the exposure time was adjusted for various temperature points). All data collections were carried out in complete darkness (the sample mounting and centering were done at room temperature prior to any further data collection, and all experiments were performed with all of the diffractometer lights permanently switched off). For details concerning photocrystallographic data-collection codes and the measurement sequence for each sample, see the Supporting Information (Tables S5 and S6). Further data processing (i.e., unit-cell determination, raw diffraction-frame integration, absorption correction, and scaling) was the same for all data sets collected. All structures were solved using an intrinsic phasing method as implemented in the *SHELXT* program^17^ and refined with the *SHELXL* program^18^ in the *OLEX2* package^19^ within the independent atom model (IAM) approximation.

### 2.3 Spectroscopy

All infrared (IR) measurements were performed using a Nicolet 5700 FTIR spectrometer (spectral resolution of 2 cm^–1^ in the range of 360–4000 cm^–1^) equipped with a closed-cycle cryostat (Oxford Optistat V01). The sample was ground, mixed with spectroscopic grade KBr, pressed into pellets (referred to as thin-film samples) via a mechanical press, and glued to the coldfinger of the cryostat using a silver-paste thermal adhesive. During measurements, the sample was kept in a vacuum inside the cryostat. Irradiation of the sample was achieved through the cryostat window using various LEDs (Thorlabs L and LP series), the central wavelengths of which covered the range from violet to red (from 385 to 735 nm). LED powers are presented in the Supporting Information (Table S2).

### 2.4 Theoretical Calculations

Isolated molecule, dimer interaction energies, and normal-mode frequencies were calculated using the density functional theory (DFT) at the DFT­(B3LYP)/6–311++G** level of theory^20–23^ using the *GAUSSIAN* package (ver. 16, rev. C.01).^24^ For harmonic-mode computations, no imaginary frequencies were found. All optimized molecular energies were corrected for the zero-point energy (ZPE). For all of the calculations performed with the *GAUSSIAN* package, the Grimme empirical dispersion correction^25,26^ modified by the Becke-Johnson damping function was applied;^27,28^ in the case of the interaction energy calculations, a correction for basis set superposition error^29,30^ (BSSE) was also included. The semiautomatic generation of input files was accomplished with the *CLUSTERGEN* program.^31^ Additionally, unit-cell parameters and atomic positions were optimized using the periodic approach implemented in the *CRYSTAL* program (ver. 17)^32^ applying the PBESOL0–3C functional and the sol-def2-mSVP basis set for all atoms.^33^

Potential-energy profiles (PEPs) calculations in the ground (S_0_) and in the lowest excited (S_1_) singlet states, as well as the approximate minimum-energy conical intersection (MECI) optimizations were performed at the time-dependent density functional theory level with the Tamm-Dancoff approximation (TDA-TDDFT).^34^ In these calculations, the B3LYP functional with the three-body Grimme dispersion correction was employed, with the 6–311++G** basis set applied for all atoms but the nickel, for which the 6–31G** basis set^35^ was used. The PEP scans have been performed with the *TURBOMOLE* suite of programs (ver. 7.1),^36^ while the *CIOPT* code^37^ interfaced with *TURBOMOLE* was used for the MECI optimization calculations.

## 3 Results and Discussion

The examined **Ni-4d** and **Ni-4d′** were synthesized using the same synthetic protocol as in our previous articles.^12,14,15^ It appears that selection of an (*N*,*N*,*O*)-donor ligand, thus the amine and ketone used in the synthesis, may affect the synthetic route, leading to an expected complex described in a series of our other articles,^14,15,38^ or to a rarer oxime-type product, as, e.g., the reported **Ni-1d′**.^12^ In the latter case, it was the use of a more basic aliphatic amine that resulted in a nontypical synthesis product. The obtained compound appeared to be an efficient photoswitch in the solid state and can be successfully isomerized from the ground-state nitro form both to the *endo*- and *exo*-nitrito isomers upon light irradiation. The direct experimental confirmation of the proposed reaction pathway was, however, challenging in this case since the *endo*- and *exo*-nitrito isomers had a very similar stability, and there was a rather low-energy barrier for transformation in both ways. Hence, for the purpose of the current study, we have decided to use similar ligands in the synthesis so as to possibly obtain both kinds of compounds, a typical product and the oxime-type one, in order to further investigate experimentally the isomerization mechanism and compare the behavior of both systems in the solid state.

Consequently, when compared to the synthesis of the previous **Ni-1d′** compound, the same aliphatic amine substrate was used, but the ketone substrate was changed from acetophenone to a more basic *N*,*N*-dimethyl acetophenone. In such a case, depending on the amount of LiNO_2_ added during the reaction, different products are obtained. These are the two desired coordination compounds, **Ni-4d** and an oxime-type **Ni-4d′**, which differ in the way the carbonyl fragment is attached to the metal center as shown in Scheme 2. In the case of **Ni-4d′**, an additional NO fragment is incorporated in the coordination sphere of Ni which is the result of the excess of nitrite salt in the reaction environment along with the basicity of the amine used. Molecular structures of **Ni-4d** and **Ni-4d′** are presented in Figure 1. Similarly to the photoswitching nickel nitro complexes previously studied by us, the metal center is coordinated by the (*N*,*N*,*O*)-donor supporting ligand and the ambidentate nitrite group, while the metal center coordination geometry is close to square-planar.^12,14^

**Figure 1:**
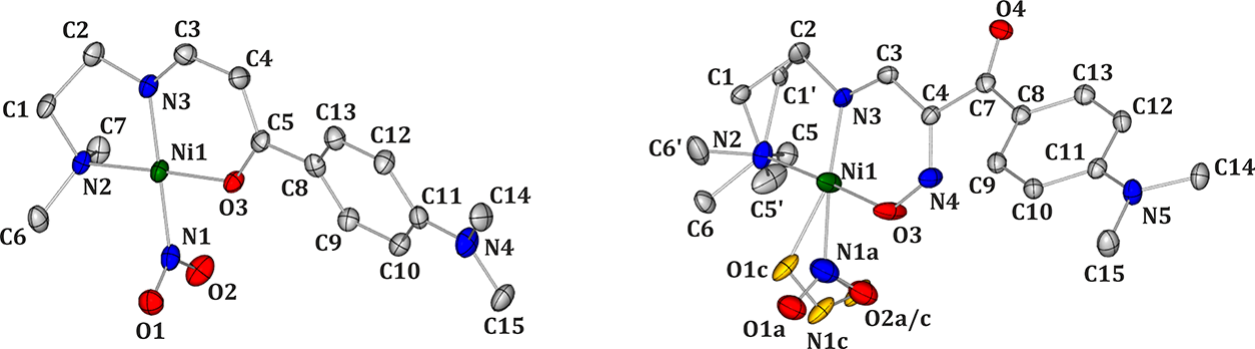
Molecular structures of **Ni-4d** (left) and **Ni-4d′** (right) derived from the X-ray diffraction data sets collected in complete darkness at 100 K (atomic thermal motion is represented as ellipsoids at the 50% probability level; hydrogen atoms are omitted for clarity; note the disorder, shown in gold, at the NO_2_ group in **Ni-4d′**).

### 3.1 Crystal Structures

**Ni-4d** crystallizes in the monoclinic *P*2_1_/c space group, with one molecule in the asymmetric unit (ASU) (Table 1). The nitrite group in the **Ni-4d** crystal structure adopts the nitro binding mode in the ground state (GS) (Figure 1).

In turn, **Ni-4d′** crystallizes in the triclinic P1̅ space group with one molecule in ASU. In the **Ni-4d′** crystal structure, the nitrite group exists in two isomeric forms, i.e., nitro and *endo*-nitrito in the GS with the nitro binding mode predominating in the crystal structure (population of ca. 70%). Multitemperature measurements of **Ni-4d′** crystal structures were performed in the 280–100 K range, showing that the *endo*-nitrito population remains generally stable (Table S7, Supporting Information). The small deviations are within experimental error.

Crystal packings of **Ni-4d** and **Ni-4d′** are different. Molecules in the crystal structure of **Ni-4d** are arranged in a herringbone-manner along the [010] direction (Figure S4, Supporting Information), which is mainly supported by C–H···π interactions and hydrogen bonds involving nitrite ligands. Furthermore, the molecules are arranged into a ribbon-like structure along the [001] direction (Figure S5, Supporting Information) which is formed mainly by C–H···π interactions and hydrogen bonds involving the nitrite ligand as in the former case. Main motif in the **Ni-4d′** structure is a dimeric plane along the [100] direction (Figure S6, Supporting Information), majorly supported by hydrogen bonds involving nitrite group as well as O3, O4, and N4 atoms from the supporting ligand along with C–H···π interactions.

**Table 1:** Selected Crystal-Structure Parameters of the Studied Complexes at 100 K Prior to Any Light Exposure (“Dark” Structures)^a^

structure/compound	**Ni-4d**	**Ni-4d′**
data set	**Ni-4d-100 K-dark-01-xtal1**	**Ni-4d-prim-100 K-dark-18-xtal1**
moiety formula	C_15_H_22_N_4_Ni_1_O_3_	C_15_H_21_N_5_Ni_1_O_4_
moiety formula mass, *M*/a.u.	365.06	394.06
crystal system	monoclinic	triclinic
space group	*P*2_1_/*c* (no. 14)	*P*1̅ (no. 2)
*Z*	4	2
*F*_000_	768	412
crystal color and shape	orange block	orange block
*T*/K	100	100
*a*/Å	11.0495(5)	7.3612(2)
*b*/Å	10.4673(4)	10.0034(3)
*c*/Å	14.7449(6)	11.8479(4)
α/°	90	85.320(3)
β/°	107.461(5)	89.590(2)
γ/°	90	72.842(3)
*V*/Å^3^	1626.79(12)	830.71(5)
*d*_calc_/g·cm^–3^	1.490	1.575
*R*[*F*] (*I* > 3σ(*I*))	4.45%	3.47%
*w*R** [*F* ^2^] (all data)	11.8%	9.03%
ϱ_res_^min/max^/e·Å^–3^	–0.41/+0.59	–0.69/+0.47
CCDC code	2433376	2433395

a For more information see Supporting Information.

Differences and similarities in the crystal packing of the examined compounds can be well illustrated and analyzed using the concept of Hirshfeld surfaces and further – fingerprint plots (Figure 2 and Table 2).^41–45^ The calculated interatomic contact contributions to the Hirshfeld surfaces generated for the **Ni-4d** and **Ni-4d′** crystal structures show that both of them are dominated by H···H interactions, which is typical for molecular crystals of organic compounds. Interestingly, the percentage contribution of these contacts is significantly smaller for **Ni-4d′**. In this case, this is balanced by notably greater contributions of the N···H and O···H type contacts. The increased impact of these interactions results from the presence of the oxime moiety (N4–O3) and the exposed carbonyl oxygen engaged in hydrogen-bond-type contacts in the crystal structure of **Ni-4d′**. This kind of interactions are naturally also formed by the nitrite group, involving its exposed oxygen atoms which contribute the most to the total number of hydrogen-bond-like contacts in both systems. From the respective fingerprint plots, it can be concluded that hydrogen bonds are stronger and more distinct in the **Ni-4d′** structure compared to **Ni-4d**. However, theoretical calculations of dimeric motifs (Tables 3 and 4 and Figures S14 and S15, Supporting Information) reveal that the nitrite group in **Ni-4d** is better stabilized by these interactions. This suggests that the strong hydrogen bonding interactions observed in the fingerprint plot for **Ni-4d′** involve other parts of the molecule rather than the nitrite group. In turn, the structure of **Ni-4d** exhibits more effective C–H···π edge-to-face interactions between the phenyl ring, the nickel-containing aromatic ring, and hydrogen atoms from methyl groups, the phenyl ring, and the nonaromatic ring as well. Similar interactions occur in the **Ni-4d′** structure; however, there is fewer of them, and the molecules adopt less favorable orientations. Based on the obtained fingerprint plots, it can also be concluded that packing efficiency is higher in the case of the **Ni-4d′** crystal structure.

**Table 2:** Interatomic Contact Contributions to the Hirshfeld Surface Generated for the **Ni-4d** and **Ni-4d′** Molecules in the GS Crystal Structures, Computed with the *CRYSTALEXPLORER* Program^40^,^a^

	Ni···H	N···O	N···C	N···H	O···O	O···C	O···H	C···C	C···H	H···H
**Ni-4d**	2.1%	0.2%	0.2%	2.9%		0.1%	23.2%		19.7%	51.5%
**Ni-4d′**	1.2%	0.7%	0.3%	10.3%	0.4%	1.2%	30.1%	0.7%	17%	38%

a Total reciprocal (i.e., X···Y and Y···X) contributions are always shown.

**Figure 2:**
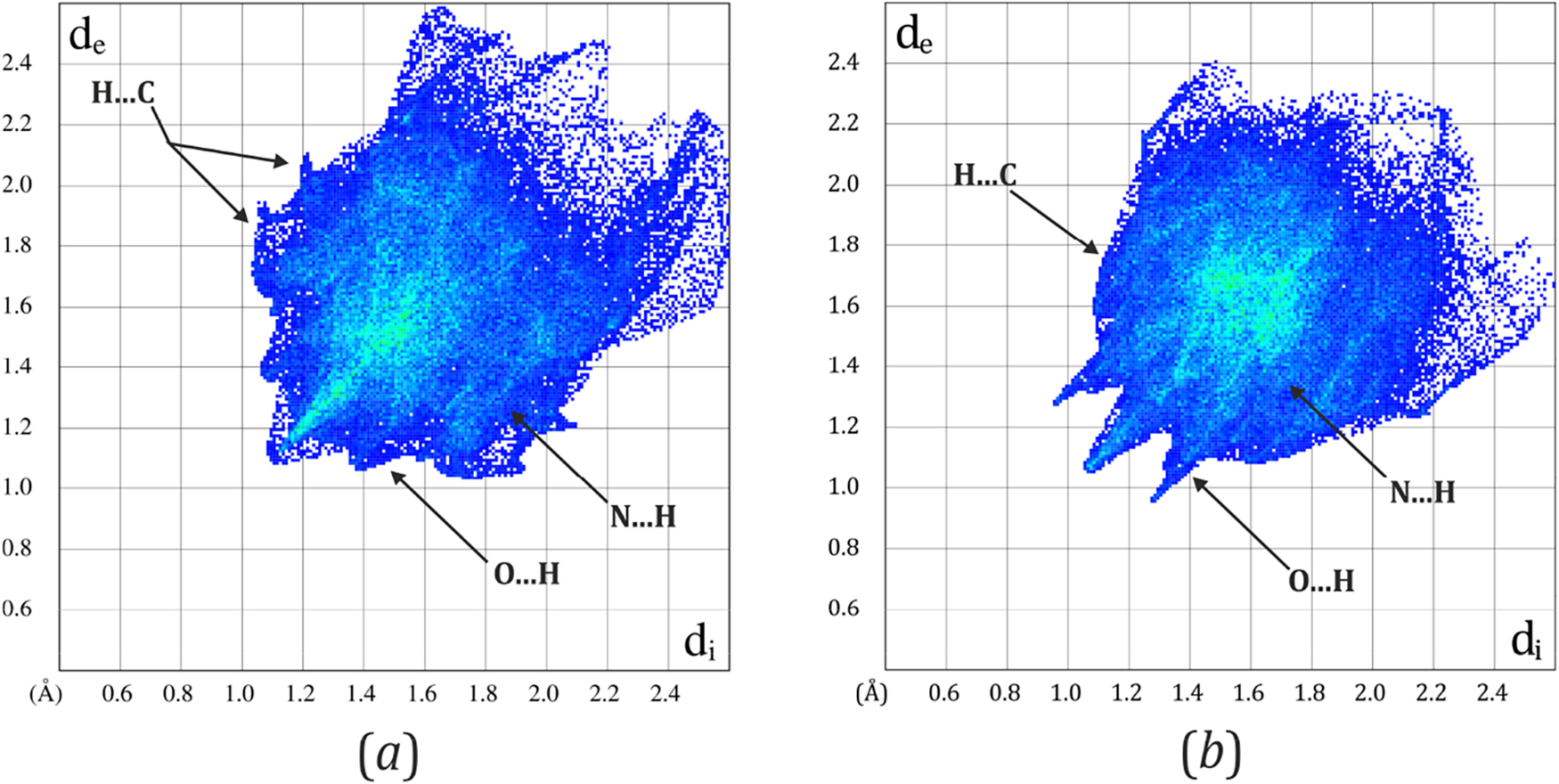
Hirshfeld fingerprint plots generated for (a) **Ni-4d** and (b) **Ni-4d′**, based on the 100 K ground-state (GS) crystal structures. The intensity of the blue color reflects the contribution of specific intermolecular interactions: the brighter the blue, the greater the contribution. The presence of diffuse regions at *d*_i_ and *d*_e_ values (normalized “internal” and “external” surface–nearest-atom distances) around 2.0 Å and higher indicates less efficient crystal packing. The more defined and sharp features are in the plots, the stronger and more directional the corresponding interactions.^39^

**Table 3:** Dimeric Motifs Engaging the NO_2_ Group and Respective Interaction Energies Based on the Experimental Ground-State Crystal Structure of the **Ni-4d** Compound (Figure S14, Supporting Information)^a^,^b^

motif	*E*_int_/kJ·mol^–1^	selected interactions	*d*_H···A_/Å	*d*_D···A_/Å	θ_D‑H···A_/Å
**D1**	–33.82	O2···H7b–C7^#1^	2.50	3.113(4)	121.74
**D2**	–107.05	O1···H1a–C1^#1^	2.53	3.293(4)	136.61
		C3···H6b–C6^#2^	2.86	3.693(5)	145.13
		C4···H6b–C6^#2^	2.80	3.500(5)	130.74
**D3**	–59.86	O1···H1b–C1^#3^	2.78	3.460(4)	128.83
		C10–H10···C3^#3^	2.92	3.693(5)	138.51

a Geometries with the X–H neutron-normalized distances were used for computations.

b Symmetry operations: (#1) −*x*, −*y*, −*z* – 1, (#2) −*x*, −*y* + 1, −*z* – 1, (#3) *x*, −*y* + 1/2, *z* – 1/2.

**Table 4:** Dimeric Motifs Engaging the NO_2_ Group and Respective Interaction Energies Based on the Experimental Ground-State Crystal Structure of **Ni-4d′** Structure (Figure S15, Supporting Information)^a^,^b^

motif	*E*_int_/kJ·mol^–1^	selected interactions	*d*_H···A_/Å	*d*_D···A_/Å	θ_D‑H···A_/Å
**D1**	–26.08	O2···H15c–C15^#1^	2.63	3.494(13)	150.42
**D1′**	–25.79	O2···H15c–C15^#1^	2.51	3.379(11)	150.54
**D3**	–49.66	C6–H6c···O2^#2^	3.84	3.740(16)	76.68
**D3′**	–37.29	N1···H5a–C5^#2^	3.84	3.813(15)	81.45
		O2···H6c–C6^#2^	2.39	2.640(11)	94.10

a Geometries with the X–H neutron-normalized distances were used for computations. **D** ***x*** denotes motifs formed by the nitro isomer, whereas **D** ***x*** **′** the ones formed by the *endo*-nitrito isomer (*x* – motif number).

b Symmetry operations: (#1) *x* – 1, *y* + 1, *z*, (#2) −*x* −2, −*y* + 2, −*z* + 1.

Since the nitrite ligand in **Ni-4d′** exists in two isomeric forms in the ground state, namely the nitro and *endo*-nitrito binding modes, intermolecular interactions formed by the nitro and *endo*-nitrito isomers can be compared. Analyzing the fingerprint plot (Figure S7, Supporting Information), it can be clearly seen that the *endo*-nitrito isomer is engaged in shorter and better-defined hydrogen bonds. However, theoretical calculations of dimeric motifs (Table 4) indicate that this does not improve the stability of this isomer, which might be due to the noticeably fewer interactions formed with oxygen in favor of interactions involving nitrogen. Still, part of the intermolecular interactions are similar for both *endo*-nitrito and nitro form. The packing for **Ni-4d′** in *endo*-nitrito form is visibly less efficient than in the case of the nitro form (Figure S7, Supporting Information).

A very useful tool in the analysis of light-induced transformations in crystals is the concept of the reaction cavity. The term and first computations were introduced by Ohashi and co-workers^46^ with a great deal of success in explaining many subtle phenomena occurring when molecules change in crystals. The method is relatively simple. It requires only “cutting out” the photoactive part from the crystal structure (in our case this would be the NO_2_ group) and analyzing the shape and volume of the artificially created “void.” Nowadays several programs can compute the crystal voids and related properties. The most well-known are *PLATON* ^47^ (grid search with the van der Waals radii criteria^48^), *MERCURY* ^49^ (“rolling-ball” method^50^), or *CRYSTALEXPLORER* (promolecule electron density cutoff criterion^51^). Here, we used *MERCURY*, with the settings identical to those in our previous work for consistency.

The reaction-cavity volumes calculated for both compounds amount to 26.3 Å^3^ for **Ni-4d** and 33.9 Å^3^ for **Ni-4d′**. Although both values are within the volume range reported for compounds exhibiting photoswitching behavior of the nitro group, the cavity in the **Ni-4d** crystal structure is at the lower limit.^14,52,53^ In the context of isomerization reaction, the cavity shape in the **Ni-4d′** crystal structure, which already hosts two linkage isomers, appears to be more favorable as it is rather evenly distributed in all directions (Figures S8 and S9, Supporting Information) providing enough space for rearrangement of the ambidentate ligand.

In view of the above, both compounds have an isomerization potential. The nitro group in both structures mainly forms hydrogen-bond-like interactions with the neighboring molecules, which, unlike strong hydrogen-bond interactions involving more electronegative atoms, may promote the linkage isomerism reaction by stabilizing the resulting isomers while also being easy to break when exposed to visible light.^52,54^ Furthermore, the reaction cavities in both crystal structures are sufficient to accommodate the ambidextrinated ligand transformation.

### 3.2 Formation and Decay of Photoinduced Linkage Isomers

#### 3.2.1 Infrared Spectroscopy

A series of solid-state infrared spectroscopic measurements were conducted for samples just after being exposed to different LEDs’ light for varying periods of time at selected temperatures. Such an approach enables characterization of the photoswitching behavior of the examined material and defines optimal conditions of the analyzed photoinduced transformation, based on intensity changes of bands assigned to the vibrations of the nitrite group. The bands’ intensity changes with irradiation time and during sample relaxation provide us also with some information about the kinetics of the process. Thermal behavior of nonirradiated samples was also investigated.

For both compounds, the respective vibration ranges for each linkage isomer under consideration are comparable. In all cases, the bands were assigned to specific vibrations based on the calculated frequencies (Table S3, Supporting Information) and available literature.^12–14^ For the nitro and *endo*-nitrito binding modes, these are bands located from 1300 to 1400 cm^–1^ and from 1070 to around 1140 cm^–1^, respectively. Upon 530 nm LED irradiation, in the IR spectrum of **Ni-4d** (Figure 3), the intensity of the 1068, 1105, and 1121 cm^–1^ bands associated with the symmetrical stretching vibrations of the *endo*-nitrito group are increasing, which is accompanied by lowering of the 1352, 1358, and 1372 cm^–1^ bands corresponding to symmetrical stretching (1352 cm^–1^) and asymmetrical stretching (1358 and 1372 cm^–1^) vibrations of the nitro isomer, respectively. The presence of the *endo*-nitrito binding mode is also evidenced by the appearance of the 1404 and 1409 cm^–1^ bands, which can be attributed to their symmetrical stretching. For **Ni-4d′**, the nitro-to-nitrito isomerization process is manifested by the intensity rise of the following bands upon 530 nm LED irradiation: 1065, 1085, 1104, and 1128 cm^–1^ related to symmetrical vibrations of the *endo*-nitrito binding mode (Figure 4). Furthermore, spectral changes accompanying this transformation are also visible in the higher vibrational frequency regions, i.e., around 1410 and 1426 cm^–1^. In turn, the nitro isomer gradual depopulation can be monitored by observation of the following bands 1321, 1330, 1361, and 1386 cm^–1^, which are assigned to asymmetrical stretching vibrations.

**Figure 3:**
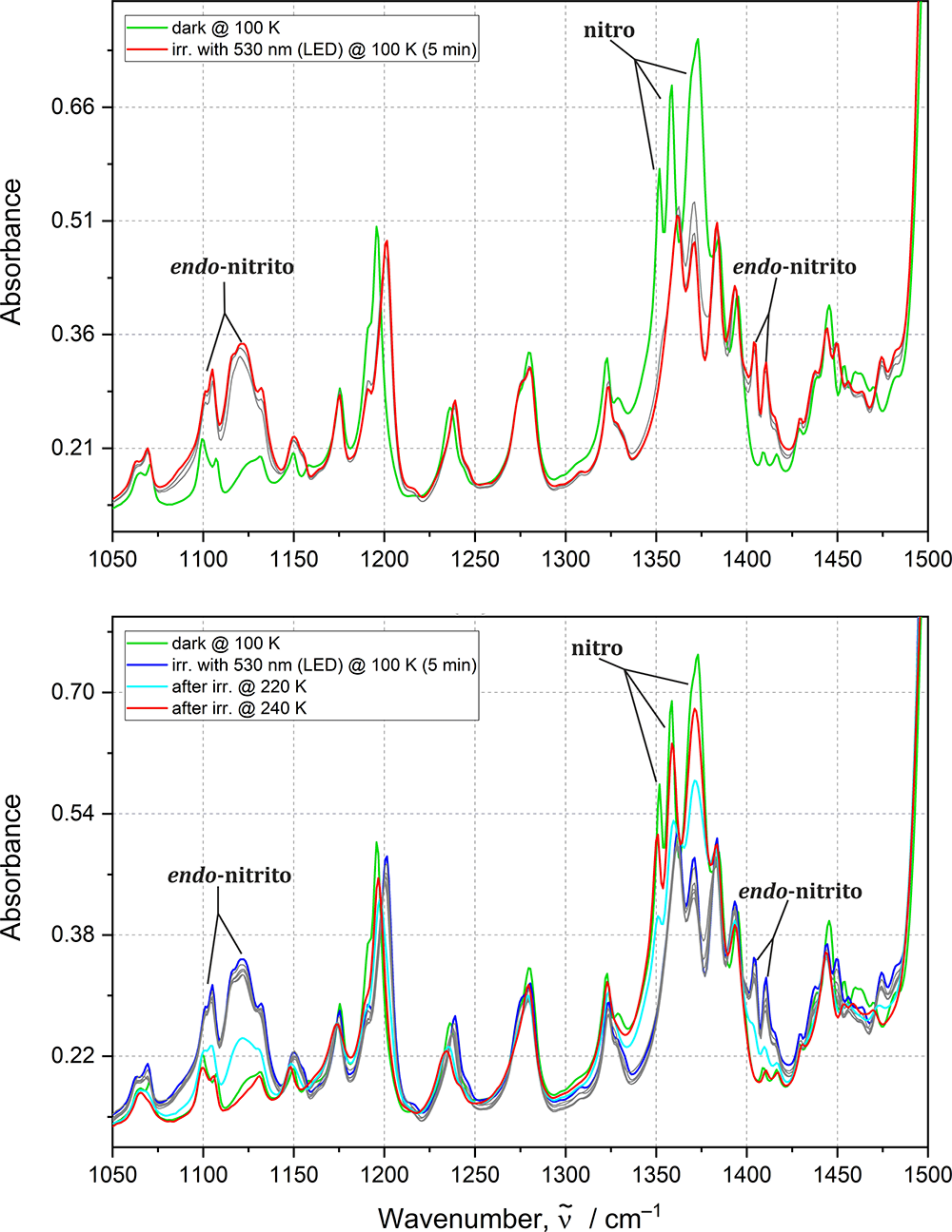
IR spectra were collected for the **Ni-4d** sample. Upper panel: before (green line) and after (red line) optimal irradiation time with 530 nm LED at 100 K for generating *endo-*nitrito form; gray lines correspond to subsequent irradiation points (1–5 min); bottom panel: before (green line) and after irradiation (blue line) with 530 nm LED at 100 K and during temperature relaxation (cyan and red line) of *endo*-nitrito form; gray lines correspond to subsequent temperature points during sample heating.

**Figure 4:**
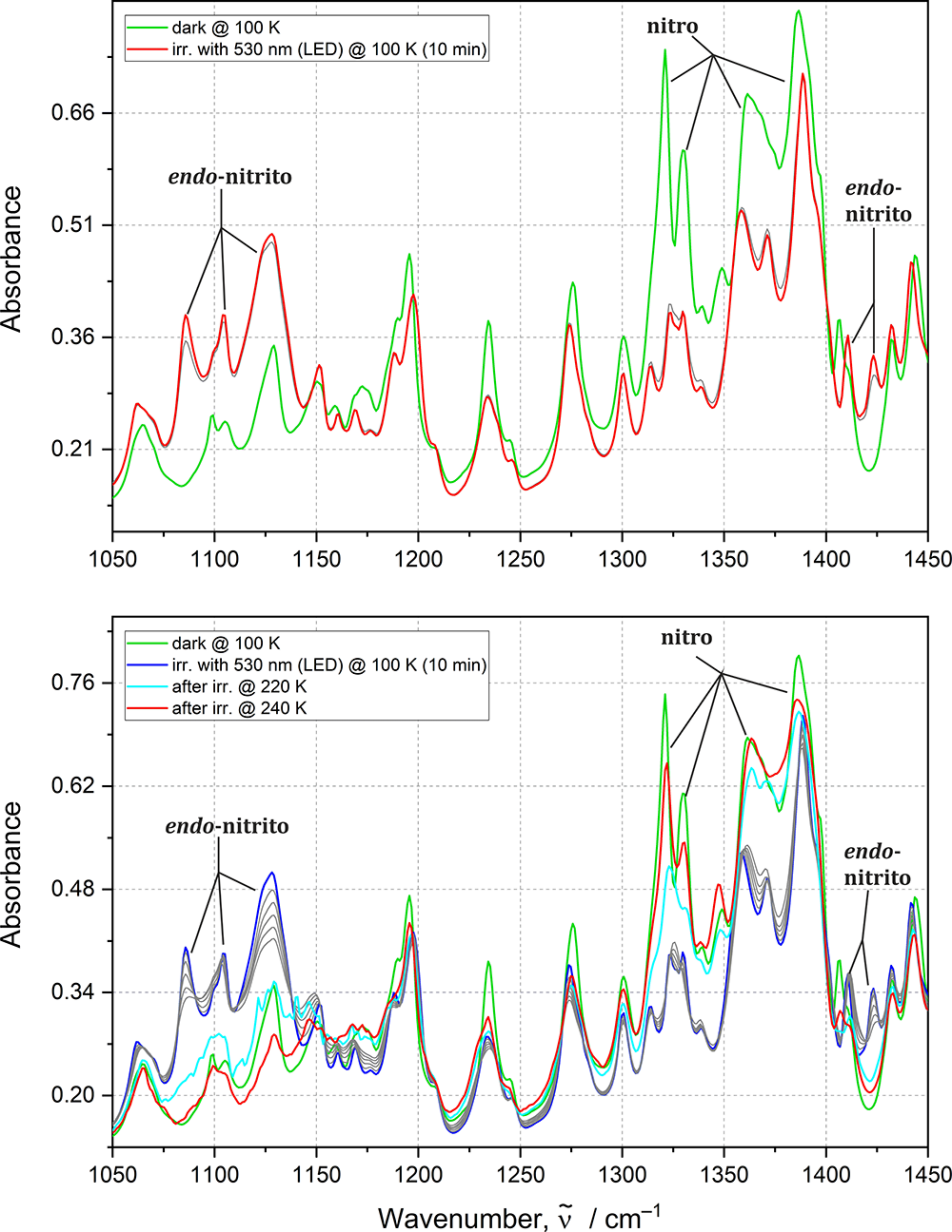
IR spectra collected for the **Ni-4d′** sample. Upper panel: before (green line) and after (red line) optimal irradiation time with 530 nm LED light at 100 K for generating the *endo-*nitrito form; gray lines correspond to subsequent irradiation point (5 min), bottom panel: before (green line), after irradiation (blue line) at 100 K, and during temperature relaxation (cyan and red line) of the *endo*-nitrito form; gray lines correspond to subsequent temperature points during sample heating.

In order to investigate the temperature effect on the samples, multitemperature measurements were conducted in the RT → 10 K range. The temperature was gradually reduced, and an IR spectrum was collected every 20 K. For both compounds, no spectral changes were observed during these experiments. However, it can be noticed that in the **Ni-4d′** sample, bands associated with the *endo*-nitrito binding mode are already visible without any irradiation, which is in agreement with the single-crystal structure of this compound.

The most significant spectral changes associated with the nitro-to-*endo*-nitrito isomerization for the examined compounds were observed after sample irradiation with a 530 nm LED light. The optimal irradiation time was determined at 100 K. It appears that maximal population of the *endo*-nitrito form is reached faster for **Ni-4d**, for which 5 min of LED irradiation, equal to 21.3 J·cm^–2^ fluence (or energy density; Table S2, Supporting Information), of thin-film sample is sufficient. In turn, the **Ni-4d′** sample requires 10 min of irradiation (42.5 J·cm^–2^ fluence) to reach the maximum conversion level. Photoinduced linkage isomers in both cases are stable up to 220 K at which their populations visibly decrease with time. At 240 K, in both cases, full relaxation to the initial state is noted.

Throughout the IR experiments, the generation of the *exo*-nitrito isomer under 530 and 660 nm LED light irradiation was detected for both **Ni-4d** and **Ni-4d′**. For **Ni-4d′**, the existence of this metastable form was clearly evidenced by the distinct appearance of 1044, 1065, and 1460 cm^–1^ bands after irradiation. These spectral changes were particularly well-defined, allowing for definitive identification of the formation of the *exo*-nitrito form. For the **Ni-4d** complex, the transition to the *exo*-nitrito form was confirmed by an increase of the 1068 and 1436 cm^–1^ bands. However, the 1068 cm^–1^ band substantially overlaps with that from the *endo*-nitrito binding mode vibrations, complicating the precise identification of the *exo*-nitrito isomer. This contrasts with the **Ni-4d′** complex, where the spectral changes allow for more reliable characterization of the isomeric transition.

The optimal irradiation wavelengths for inducing the formation of the *exo*-nitrito isomer were determined at 10 K, where this isomer can be more effectively generated. For **Ni-4d′**, short irradiation with 530 nm LED (5 min, 4.3 J·cm^–2^ fluence) produced excellent results, with clearly visible spectral bands corresponding to the *exo*-nitrito form. Notably, **Ni-4d′** requires significantly lower LED power compared to **Ni-4d** to achieve the *exo*-nitrito form with minimal admixture of the *endo*-nitrito isomer. This difference is attributed to the rapid formation of the *endo*-nitrito binding mode when **Ni-4d** is exposed to 530 nm LED light (Figures S12 and S13, Supporting Information).

Further experiments revealed that 660 nm LED light could generate even higher populations of the *exo*-nitrito form in both compounds with lower conversion to the *endo*-nitrito isomer (Figure 5). The optimal irradiation time for **Ni-4d′** was longer (1 h, 451.7 J·cm^–2^ fluence) compared to that of **Ni-4d** (Figure S12, Supporting Information). While 530 nm proved to be the energy-optimal wavelength for generating the *exo*-nitrito isomer in both complexes, the 660 nm LED provided better conditions for monitoring the isomerization process due to the slower course of the photoinduced transformation in this case.

**Figure 5:**
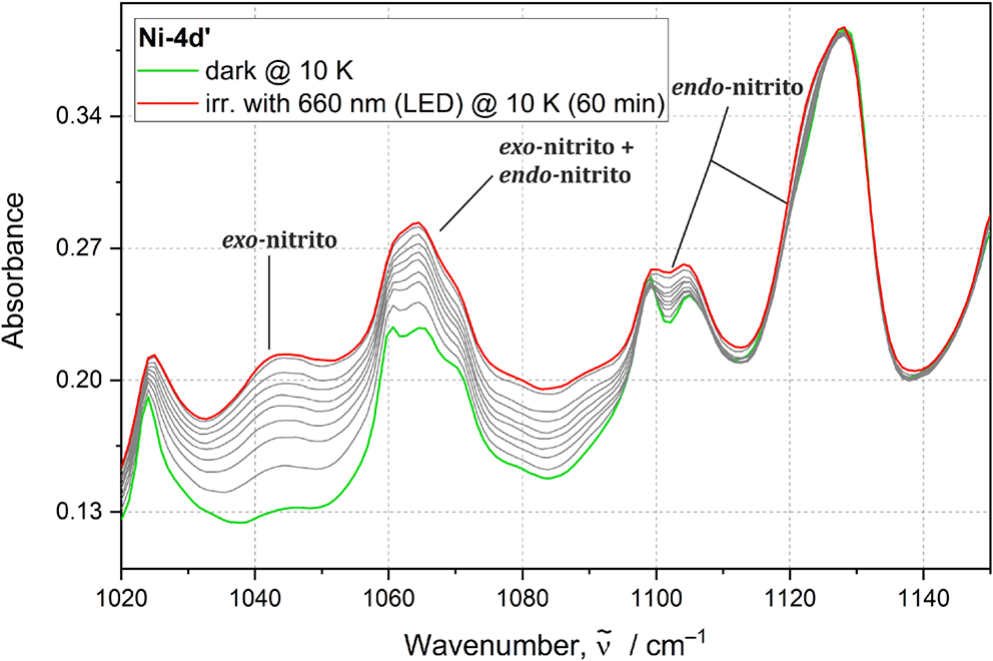
IR spectra collected before (green line) and after (red line) optimal irradiation time at 10 K for generating the *exo*-nitrito form in the **Ni-4d′** sample with 660 nm LED; gray lines correspond to subsequent irradiation points (1–60 min).

#### 3.2.2 Relaxation Kinetics of the Photoinduced Linkage Isomers

The kinetics of the relaxation of the metastable *endo*-nitrito and *exo*-nitrito isomers in the solid state was examined for both compounds (Table 5). For this purpose, a series of isothermal IR spectroscopic measurements during the relaxation process were conducted. In order to determine the decay constants (*k*) at each temperature, the first-order kinetic equation was employed. The activation energy (*E*_a_) and frequency factor (*k*_0_) were calculated based on the Arrhenius equation. The decay temperature (*T*_d_), which corresponds to the point at which *k* = 10^–3^ s^–1^, was calculated based on the obtained kinetic parameters. Despite the fact that the activation energy and frequency factor differ between the two compounds, the resulting decay temperatures, derived for both the *exo*-nitrito and *endo*-nitrito metastable forms, are comparable. For the *endo*-nitrito isomers, *T*_d_ is almost equal; hence, it can be concluded that their stability in the **Ni-4d** and **Ni-4d′** thin films is very similar. In turn, *T*_d_ determined for the *ex*o-nitrito forms differs more significantly between the studied compounds, which indicates slightly lower stability of this linkage isomer in the **Ni-4d′** solid-state sample.

**Table 5:** Kinetic Parameters of the *Endo*-Nitrito and *Exo*-Nitrito Form Relaxation Were Determined by IR Spectroscopy

	*endo*-nitrito		*exo-*nitrito	
	**Ni-4d**	**Ni-4d′**	**Ni-4d**	**Ni-4d′**
*E*_a_/kJ·mol^–1^	66(2)	77 (8)	30(3)	11.4(8)
log(*k*_0_)	13.5(5)	15.8(2)	12.77(13)	3.4(5)
*T*_d_/K	211(7)	214 (2)	100(9)	93(7)

#### 3.2.3 Photocrystallography

Having the basic knowledge about the **Ni-4d** and **Ni-4d′** crystal structures along with the results of IR spectroscopic experiments, photocrystallographic studies on single crystals were conducted. In accordance with the spectroscopic findings, single crystals were irradiated with 530 and 660 nm LED light and examined toward formation of the nitrito linkage isomers. Such experiments allow us to evaluate the actual structures of the photoinduced linkage isomers, to relatively accurately estimate their populations, and to contrast the outcomes with those deduced from the spectroscopic studies for thin-film samples. The metastable state population is determined from structural refinement of the disordered nitrite group. Based on our rough analysis (Supporting Information), the estimated standard deviations do not exceed 3%. Thus, in the following discussion, the populations are rounded and analyzed, focusing mainly on the observed trends and/or changes, taking into account possible slight deviations from the given values.

##### 3.2.3.1 Photocrystallography at 90 K

Based on the above-described kinetic measurements for the *exo*-nitrito form in the **Ni-4d** compound, it is evident that achieving a substantial population of this isomer would be a challenging task at 100 K and above. This is because the estimated lifetime of this isomer at 100 K is about 16 min, as calculated applying the Arrhenius equation with the determined and above-described kinetic parameters. Thus, during a crystallographic measurement, a significant portion of this form may undergo thermal relaxation. In turn, at 90 K, the *exo*-nitrito form’s lifetime extends to approximately 10 h, which facilitates obtaining more reliable results. The highest conversion to the *exo*-nitrito form, 25%, was reached after irradiation for 30 min with a 660 nm LED (1.86 J·cm^–2^ fluence) (Table 6). The same experiment was conducted for **Ni-4d′**—in this case, the predicted lifetime of the *exo*-nitrito form at 90 K reaches about 27 min. Nevertheless, the reaction was stopped after 30 min irradiation with a 660 nm LED (2.59 J·cm^–2^ fluence); it was possible to generate the *exo*-nitrito isomer and estimate its population to 28%, while the *endo*-nitrito population remained unchanged within the estimation error. Crystal structures obtained after irradiation are listed in Figure 6.

**Figure 6:**
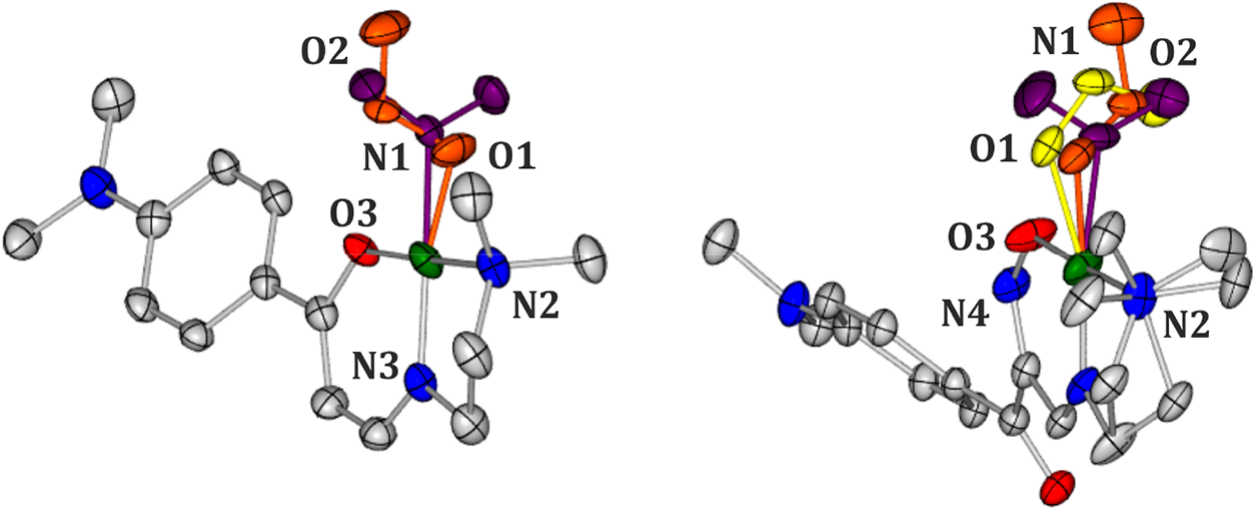
Molecular structures of **Ni-4d** (left) and **Ni-4d′** (right) derived from the X-ray diffraction data sets collected after 30 min of irradiation with 660 nm LED at 90 K. Note the disorder on the NO_2_ site (nitro isomer - violet color, *exo*-nitrito - orange, *endo*-nitrito - yellow, atomic thermal motion is represented as ellipsoids at the 50% probability level, hydrogen atoms are omitted for clarity, only selected atom labels are shown - disorder labels, A/B/C are not shown for clarity, atoms N1, O1, N2 are approximately shown).

**Table 6:** Populations of Linkage Isomers before and after Irradiation with a 660 nm LED for 30 Min at 90 K for **Ni-4d** and **Ni-4d′**

	**Ni-4d**			**Ni-4d′**		
irradiation time	nitro	*endo-*nitrito	*exo*-nitrito	nitro	*endo-*nitrito	*exo*-nitrito
before irradiation	100%	0%	0%	69%	31%	0%
after 30 min irradiation	75%	0%	25%	43%	29%	28%

##### 3.2.3.2 Photocrystallography at 100 K

The ground-state structure of **Ni-4d** at 100 K contained exclusively the nitro form. In order to determine the optimal irradiation time to generate the metastable states, the sample was irradiated within three time steps with 530 nm LED (Figure 7 and Table 7). After 1 h (1.05 J·cm^–2^ fluence), a population of 35% of the *endo*-nitrito and 8% of the *exo*-nitrito linkage isomer was generated. During the subsequent 1 h of light exposure (2.10 J·cm^–2^ fluence), the *exo*-nitrito isomer completely vanished, while the *endo*-nitrito binding mode’s population increased to 74%. The maximal conversion to this latter form was achieved after 2 additional hours of irradiation (4.21 J·cm^–2^ fluence) and reached about 92%. Regarding the relaxation of the *endo*-nitrito linkage isomer in **Ni-4d** crystals, it appears that up to 180 K, no population changes are observed. At 200 K, there is a small reduction of the *endo*-nitrito (by 7%) population, which sharply decreases to 6% at 220 K. At 240 K, complete relaxation to the ground-state structure was noted.

**Figure 7:**
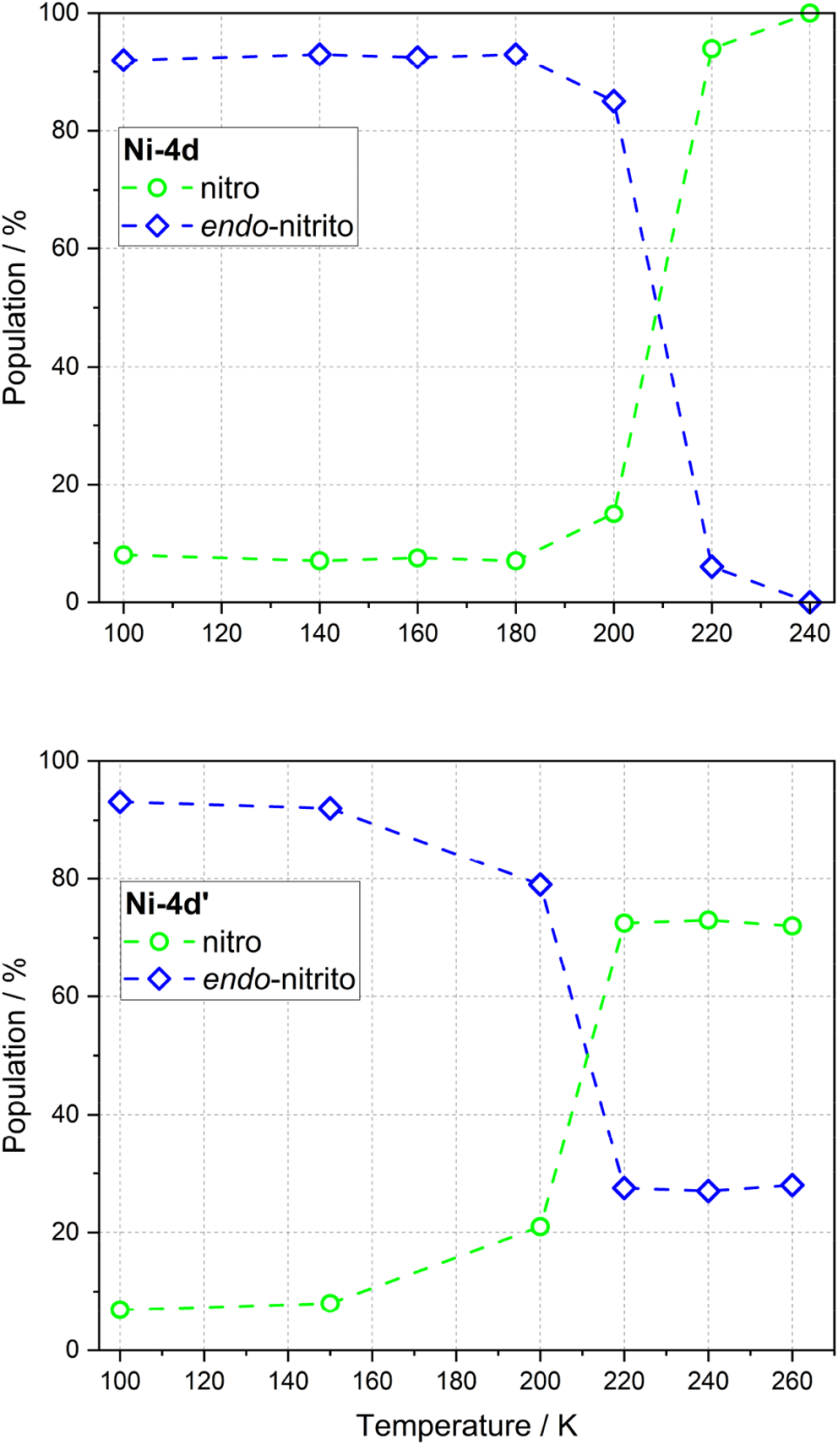
Average populations estimated for each isomer (green line - nitro, blue line - *endo*-nitrito) after irradiation with 530 nm LED at 100 K and during temperature relaxation. Upper panel presents results obtained for **Ni-4d** while bottom panel presents results obtained for **Ni-4d′**. These population values are averaged, accounting for the concurrent decay occurring during the data-collection period at each temperature point.

**Table 7:** Populations of Different Linkage Isomers before and during Subsequent Steps of Irradiation with 530 nm LED at 100 K for **Ni-4d** and **Ni-4d′**

	**Ni-4d**			**Ni-4d′**		
irradiation time	nitro	*endo*-nitrito	*exo*-nitrito	nitro	*endo*-nitrito	*exo*-nitrito
before irradiation	100%	0%	0%	70%	30%	0%
after 1 h irradiation	57%	35%	8%	^a^	^a^	^a^
after 2 h irradiation	26%	74%	0%	19%	82%	0%
after 4 h irradiation	8%	92%	0%	7%	93%	0%

a Data not collected after this irradiation time.

Similar investigations were conducted for the **Ni-4d′** crystal structure (Figure 7 and Table 7). As already mentioned, in the ground-state structure at 100 K, it consists of ca. 30% of the *endo*-nitrito form and ca. 70% of the nitro form. The crystal was irradiated with a 530 nm LED in two time steps, each lasting for 2 h. The initial 2 h light exposure (16.94 J·cm^–2^ fluence) led to an increase of the *endo*-nitrito population to 82%, while during the subsequent 2 h of irradiation (33.88 J·cm^–2^ fluence), the population of the *endo*-nitrito isomer further increased reaching 93%. Due to high population of the *endo*-nitrito isomer in the ground state, the relaxation of the photoinduced *endo*-nitrito form is particularly interesting. The population of *endo*-nitrito isomer remains stable up to 150 K and decreases to 79% at 200 K. As the temperature rises to 220 K, the population further diminishes to 28%, reaching a thermally invariant state at this point—the population of the *endo*-nitrito isomer remains constant at higher temperatures. Crystal structures obtained after irradiation are listed in Figure 8.

**Figure 8:**
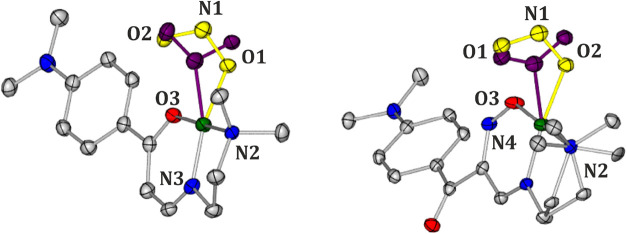
Molecular structures of **Ni-4d** (left) and **Ni-4d′** (right) derived from the X-ray diffraction data sets collected after 4 h of irradiation with 530 nm LED at 100 K. Note the disorder on NO_2_ group (nitro isomer - violet color, *exo*-nitrito - orange, *endo*-nitrito - yellow, atomic thermal motion is represented as ellipsoids at the 50% probability level, hydrogen atoms are omitted for clarity, only selected atom labels are shown - disorder labels, A/B/C are not shown for clarity, atoms N1, O1, N2 are approximately shown).

### 3.3 Mechanism of the Nitro-to-*Endo*-Nitrito Transformation

As previously described, irradiation of the samples with a 530 nm LED can result in the generation of both *endo*- and *exo*-nitrito forms. For both compounds, the isomerization reaction is notably slower at 10 than at 100 K. Thus, IR experiments with gradual irradiation with 530 nm LED at 10 K were conducted in order to shed light on the photoisomerization reaction mechanism.

Formation of both photogenerated binding modes is easier to trace for **Ni-4d′**; since, in this case, it is possible to differentiate bands related to both metastable forms. It can be clearly seen that after the first cycle of irradiation for 5 min, the bands associated with the *exo*-nitrito and *endo*-nitrito form appear (Figure 9). After the second irradiation step (additional 10 min of irradiation), there is a noticeable decrease of the *exo*-nitrito population with a concurrent increase in the population of the *endo*-nitrito linkage isomer. During further light exposure (for 20, 30, 60, 90, and 120 min), the *exo*-nitrito isomers’ population gradually diminishes until it completely disappears, while the population of the *endo*-nitrito binding mode continuously increases (Figure 10).

**Figure 9:**
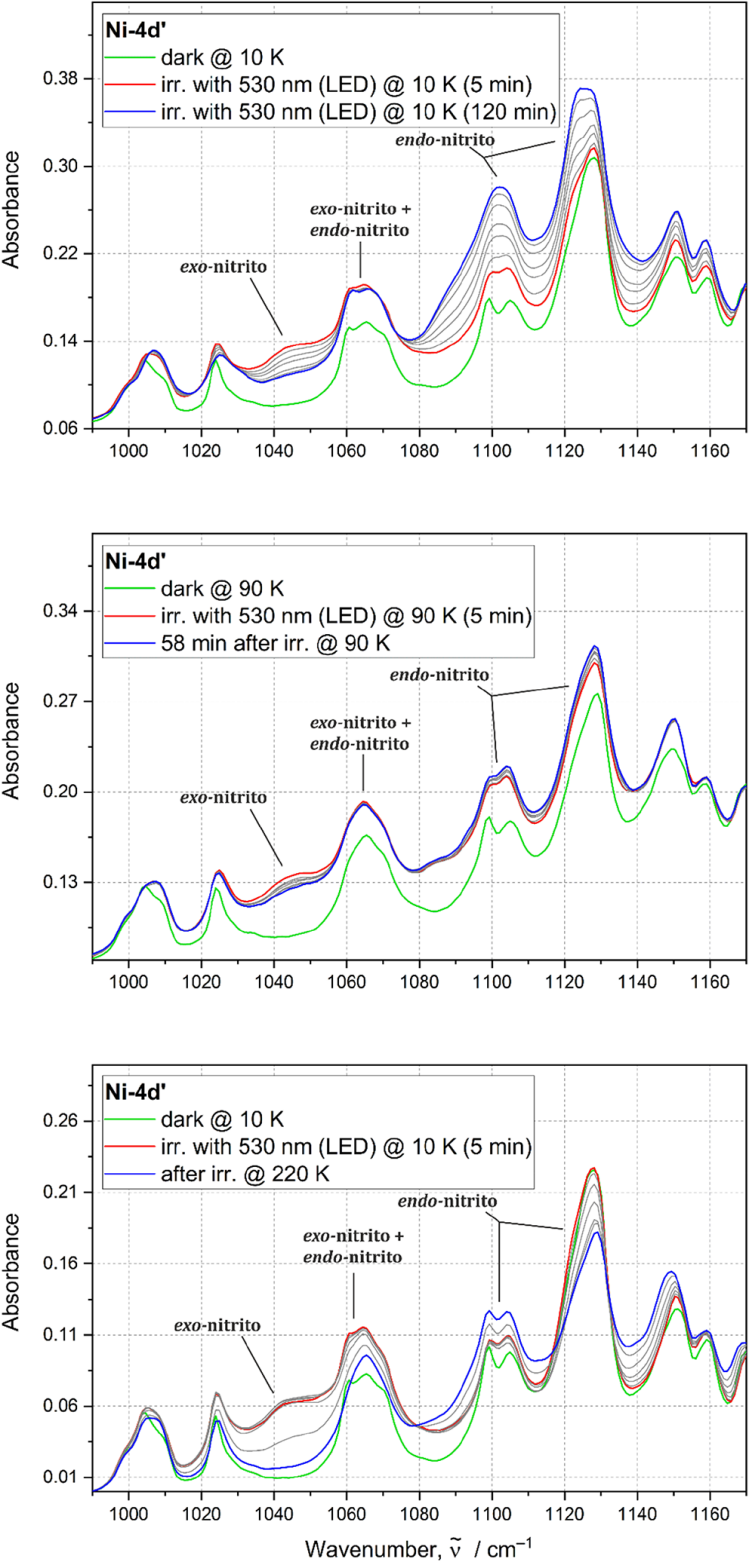
IR spectra collected for the **Ni-4d′** sample during: (i) top panel: subsequent cycles of irradiation with 530 nm LED at 10 K, (ii) middle panel: *exo*-nitrito isomer relaxation over time at 90 K, (iii) bottom panel: thermal relaxation of the *exo*-nitrito isomer in the 10–220 K temperature range. Green line indicates the initial state, red line represents spectra collected after irradiation, and blue line signifies the final state. Gray lines represent spectra collected between irradiation and final state.

**Figure 10:**
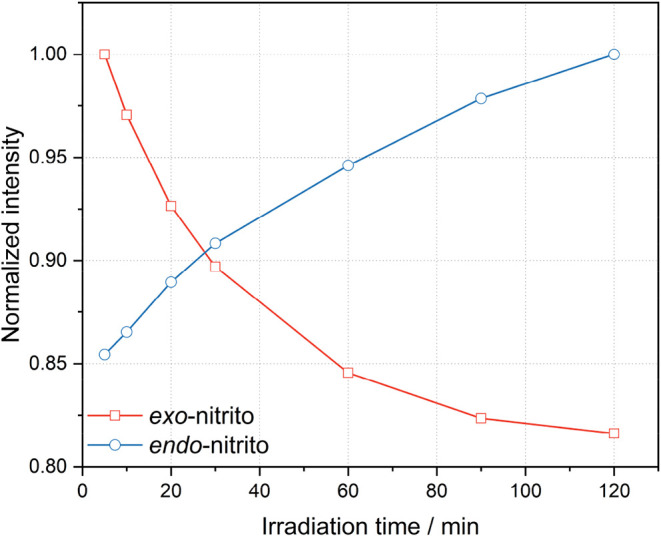
Intensity evolution of the characteristic bands for the *exo*- and *endo*-nitrito binding modes during irradiation of **Ni-4d′** with 530 nm LED at 10 K.

The reaction mechanism can also be investigated via tracing of the *exo*-nitrito state thermal relaxation (Figure 11). Thus, after reaching maximal intensity of the band associated with the *exo*-nitrito form at 10 K with 530 nm, the sample was heated and IR spectra were collected with a 20 K step until the complete disappearance of the *endo*-nitrito isomer at 220 K. Additionally, it was observed that at 90 K, the *exo*-nitrito binding mode, once generated, transforms into the *endo*-nitrito isomer in time when the irradiation is stopped. This clearly suggests that the *exo*-nitrito isomer converts directly to the *endo*-nitrito binding mode by thermal relaxation.

**Figure 11:**
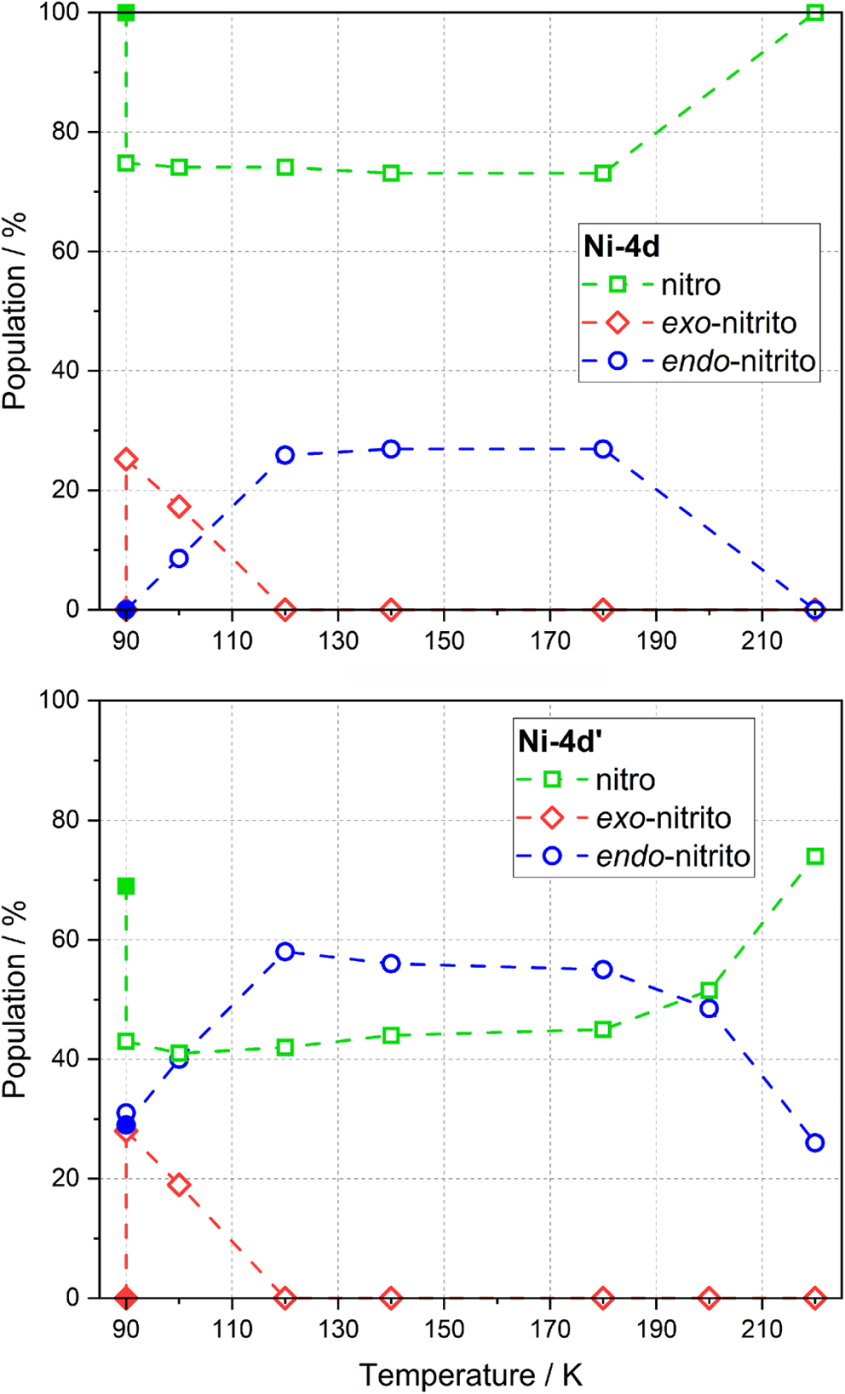
Populations estimated for each isomer (green line - nitro, red line - *exo*-nitrito, blue line - *endo*-nitrito) before irradiation at 90 K (filled points), after irradiation with 660 nm LED for 30 min at 90 K, and during temperature relaxation. Upper panel presents results obtained for **Ni-4d** compound while bottom panel presents results obtained for the **Ni-4d′** compound.

In the IR experiments for **Ni-4d**, an identical pattern was observed under irradiation with a 530 nm LED at 10 K and during thermal and time relaxation of the *exo*-nitrito form.

Finally, related photocrystallographic experiments were conducted in order to verify the applicability of spectroscopic observations to single-crystal samples. Experimental conditions were selected based on kinetic considerations and the available lowest temperature for the X-ray diffraction studies at our home laboratory. Hence, single crystals of **Ni-4d** and **Ni-4d′** were irradiated with a 660 nm LED light for 30 min at 90 K so as to generate the *exo*-nitrito binding mode. 25% of this isomer was generated in the case of the **Ni-4d** system, whereas for **Ni-4d′**, such population reached 29% (the admixture of around 30% of the *endo*-nitrito form remained the same as for the ground-state structure). In order to verify the isomerization mechanism, i.e., the *endo*-nitrito isomer formation through the *exo*-nitrito form as an intermediate photoproduct, the X-ray diffraction measurements were conducted during heating at the following temperatures: 100, 120, 140, 180, and 220 K. This way the relaxation channel of the process was probed. Based on these experiments, it is clearly visible that during relaxation, the *exo*-nitrito form fully transforms to the *endo*-nitrito linkage isomer. Starting at 100 K, partial conversion of the *exo*-nitrito form to the *endo*-nitrito binding mode was observed. In the **Ni-4d** crystal, population of the *exo*-nitrito species decreased to about 16% concurrently generating 9% of the *endo*-nitrito form. Similarly, in **Ni-4d′**, the relaxation of the *exo*-nitrito isomer to 19% resulted in 12% increase of the *endo*-nitrito form’s population. Full conversion to the *endo*-nitrito isomer occurred at 120 K in both systems and its population remained stable up to 220 K, at which the *endo*-nitrito isomer fully relaxed to the nitro form in **Ni-4d**, while in **Ni-4d′**, the population of this isomer decreased to 26%—which is comparable to its initial value.

The theoretical modeling of the mechanism coincides with the results obtained in our previous paper concerning the **Ni-1d′** compound in the solid state. First, to get a notion of a possible source of differences in the interisomer transformation dynamics observed in the electronic ground state for **Ni-4d** and **Ni-4d′**, we had a look at the S_0_ potential-energy profiles obtained for respective isolated molecules. The S_0_ profiles between the nitro, *exo*-nitrito, and *endo*-nitrito forms, calculated along the O–N–Ni angle (nitro → *exo*-nitrito), and O–N–O–Ni torsion angle (*exo*-nitrito → *endo*-nitrito) coordinates, are shown in Figure 12. One can notice that, in both cases, the barriers for the nitro → *exo*-nitrito transformation, estimated to ca. 0.936 (90.3 kJ·mol^–1^) and 0.910 eV (87.8 kJ·mol^–1^) for **Ni-4d** and **Ni-4d′**, respectively, significantly exceed the corresponding *exo*-nitrito → *endo*-nitrito reaction barriers of 0.321 (31.0 kJ·mol^–1^) and 0.293 eV (28.3 kJ·mol^–1^). Moreover, the slightly smaller values obtained for the **Ni-4d′** system stay in line with the observed lower thermal stability of its nitrito forms, on the one hand, and the faster *exo*-nitrito to *endo*-nitrito transformation, on the other.

**Figure 12:**
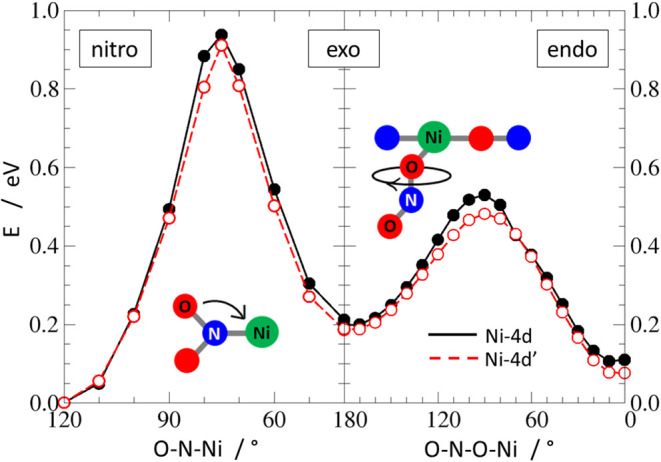
Potential-energy profiles (PEPs) calculated for the S_0_ state along the O–N–Ni valence angle (nitro → *exo*-nitrito, left panel), and the O–N–O–Ni torsion angle (*exo*-nitrito → *endo*-nitrito, right panel), respectively (black curve - **Ni-4d**, red curve - **Ni-4d′**).

In the next step, to investigate the possible mechanism of the photoinduced nitro → *exo*-nitrito transformation expected to be the primary photoswitching channel in the studied compounds, we calculated the S_1_-optimized potential-energy profiles (Figure 13) along the O–N–Ni angle: a reaction coordinate used also in the S_0_ PEP calculations. Both for **Ni-4d** and **Ni-4d′**, it can be observed that after the S_0_ → S_1_ excitation, initially the system ends up in a rather flat S_1_ potential-energy surface area, protected from the reactive transformation by a small energy barrier of 0.061 (5.9 kJ·mol^–1^) and 0.054 eV (5.2 kJ·mol^–1^) for **Ni-4d** and **Ni-4d′**, respectively. At the same time, the expected kinetic energy excess gained at the Franck–Condon vicinity due to the initial relaxation from the respective vertically excited GS structures amounts for **Ni-4d** and **Ni-4d′** to 0.392 (37.8 kJ·mol^–1^) and 0.403 eV (38.9 kJ·mol^–1^). Thus, it can be expected that after some time spent in the Franck–Condon area, the excited nitro isomer may easily reach a potential-energy surface region characterized with a very small S_1_–S_0_ energy gap and, eventually, relax through a nearby, energy-accessible conical intersection. Such minimum-energy conical intersection points have been identified for both studied systems; their geometrical structures can be found in the Supporting Information (Figure S18). Energy-wise, both found MECIs lie below the scanned S_1_ profiles, characterized by relative energies of 1.780 (171.7 kJ·mol^–1^) and 1.848 eV (178.3 kJ·mol^–1^) for **Ni-4d** and **Ni-4d′**, respectively.

**Figure 13:**
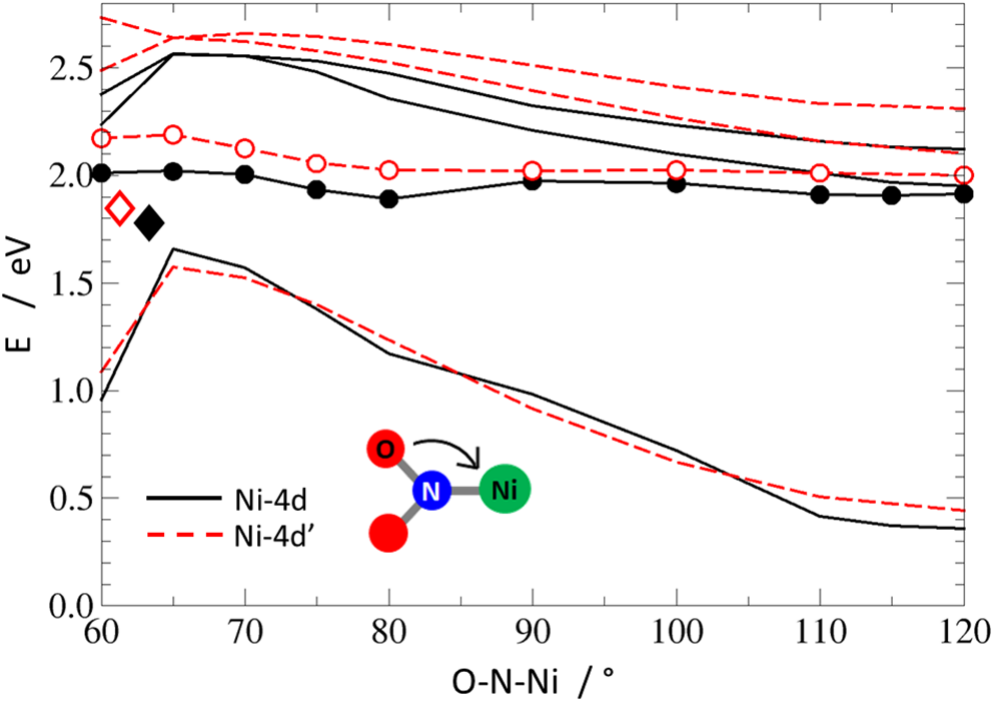
Potential-energy profiles along the O–N–Ni reaction coordinate optimized in the S_1_ state for the **Ni-4d** (black lines) and the **Ni-4d′** (red lines) systems. The optimized state in each set has been marked with circles: full for **Ni-4d** and empty for **Ni-4d′**, while the other states, calculated vertically on the S_1_ geometries, are marked with solid/dashed lines for **Ni-4d**/**Ni-4d′**, respectively. The black-full/red-empty diamonds mark positions of the optimized MECI points of **Ni-4d** and **Ni-4d′**.

At the same time, we note that no direct photoreaction path between the nitro and *endo*-nitrito isomers was established in the course of the present study, which is in agreement with our previous study on a closely related system.^12^

## 4 Molecular Stability and Intermolecular Interactions Analysis

To support the experimental findings and possibly gain deeper insights into the examined processes, structure optimizations and interaction energy computations were executed. Isolated-molecule geometry optimizations were performed in the *GAUSSIAN* program, as well as crystal-structure optimizations in *CRYSTAL*.^32^ The obtained results are summarized in Tables 8 and 9. Based on the calculations and experimental outcomes, it is evident that the nitro form constitutes the most stable linkage isomer in the case of both compounds, whereas the *exo*-nitrito binding mode itself is the least energetically favored one. The stability of the *exo*- and *endo*-nitrito isomers is comparable for the **Ni-4d** and **Ni-4d′** compounds as far as the isolated molecules are concerned. However, after optimizing the whole crystal structure, it becomes clear that packing and intermolecular interactions play an important role in the stability of different forms of the nitrite ligand, especially in the case of the **Ni-4d** crystal structure. The calculated energy differences between the considered linkage isomers become more significant in this case, which is in contrast to **Ni-4d′** for which they remain similar to the ones calculated for the isolated molecules. The minor role of intermolecular interactions for **Ni-4d′**, thus very similarly energetically stabilized nitro and *endo*-nitrito isomers in the crystal environment (<6 kJ·mol^–1^ energy difference), supports their coexistence in the crystal structure. In turn, the deformation energies provided in Table 9 appear significantly larger for **Ni-4d′**—this might, to some extent, explain a greater difficulty in forming crystals which was noted during crystallization in the experimental process.

**Table 8:** Energy Differences (*E*_diff_) between the Ground (Nitro) and Metastable (*Exo*- and *Endo*-Nitrito) Linkage Isomers Computed for the Optimized Isolated-Molecule Geometries and for the Molecular Geometries from the *CRYSTAL*-Optimized **Ni-4d** and **Ni-4d′** Crystal Structures

	*E*_diff_/kJ·mol^–1^			
	**Ni-4d**		**Ni-4d′**	
isomer	isolated mol.	*CRYSTAL*	isolated mol.	*CRYSTAL*
nitro	0.00	0.00	0.00	0.00
*endo*-nitrito	8.16	16.79	5.78	5.66
*exo*-nitrito	16.44	30.93	17.69	17.50

**Table 9:** Deformation Energy (*E*_def_) between Optimized Isolated Molecule and the Molecule Optimized in the Crystal Environment (*E*_def_ = *E*_cry_ – *E*_isol_) Presented for the Nitro, *Endo*-Nitrito, and *Exo*-Nitrito Isomers Both for **Ni-4d** and **Ni-4d′**

	*E*_def_/kJ·mol^–1^	
isomer	**Ni-4d**	**Ni-4d′**
nitro	21.12	28.74
*endo*-nitrito	17.06	29.22
*exo*-nitrito	22.54	30.44

Additionally, interaction energies between dimeric motifs’ components involving different ambidentate ligand’s binding modes were analyzed (Tables 10 and 11, and Figures S14 and S15, Supporting Information). Generally, the nitrite ligand is engaged mostly in weak, hydrogen-bond-type interaction, such as O···H–C and N···H–C. The dimeric motifs in the **Ni-4d** structure are overall better energetically stabilized than those in **Ni-4d′**. The energies and interactions of the corresponding dimeric motifs of **Ni-4d** noticeably vary between isomeric forms in some cases. Primarily, the nitro isomer in the **Ni-4d** structure creates an additional interaction relative to the *exo-* and *endo*-nitrito isomers, which is present in the **D4** motif. The *exo*-nitrito form is best stabilized in dimer **D6**, where its interaction energy is lower by ca. 20 kJ·mol^–1^ when compared to other isomers, which results from the formation of a hydrogen bond with the nitrogen atom from the NO_2_ group. All of the isomers are similarly stabilized in motif **D7**. In motif **D8**, each isomer is engaged in different intermolecular interactions, which results in noticeable differences in their energies with the nitro group being the most stabilized and the *exo*-nitrito form the least. In turn, the stabilization energies for the corresponding dimeric motifs formed by various isomers in **Ni-4d′** are quite similar. In motif **D1**, the *exo*-nitrito form is slightly less stabilized due to the formation of hydrogen bonds with the nitrogen instead of the oxygen atom in the case of the remaining isomers. However, **D2** and **D8** engaging the *exo*-nitrito form are characterized by the lowest energy. The overall stabilization energies of the *endo*-nitrito and nitro forms are very similar, which, combined with the small energy gap between the optimized molecules of these isomers, calculated both for the isolated molecule and within the crystal environment (Table 8), could explain the initial high population of the *endo*-nitrito isomer in the ground state of the **Ni-4d′** crystal.

**Table 10:** Dimeric Motifs Engaging the NO_2_ Group and the Respective Interaction Energies Based on the *CRYSTAL*-Optimized Crystal Structures for Each Linkage Isomer in the **Ni-4d** Structure (Figure S14, Supporting Information)^a^,^b^

motif	*E*_int_/kJ·mol^–1^	selected interactions	*d*_H···A_/Å	*d*_D···A_/Å	θ_D‑H···A_/Å
**D1**	–30.69	O2···H7b–C7^#1^	2.50	3.113	121.74
**D2**	–92.91	O1···H1a–C1^#2^	2.53	3.293	136.61
		C3···H6b–C6^#2^	2.86	3.693	145.13
		C4···H6b–C6^#2^	2.80	3.500	130.74
**D2′**	–91.90	O1···H1b–C1^#2^	2.46	3.193	132.74
		C3···H6c–C6^#2^	2.87	3.717	147.95
		C4···H6c–C6^#2^	2.82	3.497	128.44
**D2**″	–112.83	N1···H1a–C1^#2^	2.52	3.36	146.72
		C4···H6b–C6^#2^	2.79	3.507	131.86
		C5···H6b–C6^#2^	2.73	3.454	132.87
**D3**	–37.30	C11···H6a–C6^#3^	3.00	3.638	125.18
		N4···H6a–C6^#3^	2.85	3.729	152.03
**D3′**	–36.76	C11···H6a–C6^#3^	2.96	3.604	125.44
		N4···H6a–C6^#3^	2.78	3.663	152.59
**D3**″	–35.25	C14–H14b···O1^#3^	3.17	3.772	122.03
		C11···H6a–C6^#3^	3.00	3.638	125.18
		C12···H6a–C6^#3^	3.57	4.049	113.08
**D4**	–60.96	O1···H1b–C1^#4^	2.78	3.460	128.83
		C10–H10···C3^#4^	2.92	3.693	138.51
**D4′**	–55.27	N1···H1a–C1^#4^	2.63	3.325	129.25
		N1···H2b–C2^#4^	2.70	3.191	112.55
		C10–H10···C3^#4^	2.92	3.772	147.92
**D4**″	–48.73	O2···H1b–C1^#4^	2.20	3.014	141.56
		C10–H10···C3^#4^	2.92	3.715	141.26

a **D** ***x*** denotes motifs formed by the nitro isomer, **D** ***x*** **′** formed by the *endo*-nitrito isomer, and **D*x*″** formed by the *exo*-nitrito isomer (*x* – motif number).

b Symmetry operations: (#1) −*x*, −*y*, −z – 1, (#2) −*x*, −*y* + 1, −*z* – 1, (#3) *x* – 1, −*y* + 1/2, *z* – 1/2, (#4) *x*, −*y* + 1/2, *z* – 1/2.

**Table 11:** Dimeric Motifs Engaging the NO_2_ Group and the Respective Interaction Energies Based on the *CRYSTAL*-Optimized Crystal Structures for Each Linkage Isomer in the **Ni-4d′** Structure (Figure S15, Supporting Information)^a^,^b^

motif	*E*_int_/kJ·mol^–1^	selected interactions	*d*_H···A_/Å	*d*_D···A_/Å	θ_D‑H···A_/Å
**D1**	–25.67	O2···H15c–C15^#1^	2.63	3.494	150.42
**D1′**	–25.87	O2···H15c–C15^#1^	2.50	3.38	151.2
**D1**″	–24.79	N1···H15c–C15^#1^	2.63	3.494	150.42
**D2**	–86.16	O3···H1a–C1^#2^	2.62	3.191	118.39
		N4···H2b–C2^#2^	2.67	3.187	113.93
		C9···H2b-C2^#2^	2.81	3.670	148.95
		C14–H14c···O4^#2^	2.32	3.136	142.89
**D2′**	–84.53	O3···H1a–C1^#2^	2.62	3.191	118.39
		N4···H2b–C2^#2^	2.67	3.187	113.93
		C9···H2b-C2^#2^	2.81	3.670	148.95
		C14–H14c···O4^#2^	2.32	3.136	142.89
**D2**″	–87.34	O2···H6a–C6^#2^	2.50	3.415	160.37
		O3···H1a–C1^#2^	2.62	3.191	118.39
		N4···H2b–C2^#2^	2.67	3.187	113.93
		C9···H2b-C2^#2^	2.81	3.670	148.95
		C14–H14c···O4^#2^	2.32	3.136	142.89
**D3**	–47.27	C6–H6c···O2^#3^	3.84	3.740	76.68
**D3′**	–46.77	N1···H5a–C5^#3^	3.84	3.813	81.45
		O2···H6c–C6^#3^	2.39	2.640	94.10
**D3**″	–51.00	O2···H5a–C5^#3^	2.88	2.920	82.94
		O2···H6c–C6^#3^	2.71	3.481	91.35

a **D** ***x*** denotes motifs formed by the nitro isomer, **D** ***x*** **′** formed by the *endo*-nitrito isomer, and **D*x*″** formed by the *exo*-nitrito isomer (*x* – motif number).

b Symmetry operations: (#1) *x* – 1, *y* + 1, *z*, (#2) *x* + 1, *y*, *z*, (#3) −*x* – 2, −*y* + 2, −*z* + 1.

## 5 Conclusions

Two related square-planar nickel­(II) complexes—**Ni-4d** and **Ni-4d′**—were designed in order to investigate the mechanism of photoinduced nitro-to-*endo*-nitrito transformation. For both **Ni-4d** and **Ni-4d′**, we were able to generate and experimentally observe all three forms of the nitrite ligand—nitro, *endo*-nitrito, and *exo*-nitrito isomers—which made these complexes promising for tracking the photoisomerization pathway. For both systems, the *endo*-nitrito isomer can be generated most efficiently using 530 nm LED light with nearly 100% conversion at 100 K. This isomer showed comparable stability for both compounds (*T*_d_ ∼ 210–215 K) in the solid state. In turn, the *exo*-nitrito isomer is formed under 530 or 660 nm LED light irradiation. However, its observation is facilitated at temperatures below 100 K, due to its lower thermal stability (*T*_d_ amounts to 100 K for **Ni-4d** and 93 K for **Ni-4d′**). The maximal obtained populations of this form based on the photo-XRD experiments amounted to ca. 25% at 90 K in single crystals of both compounds when irradiated with 660 nm LED light. The lower stability of the *exo*-nitrito isomer compared to the *endo*-nitrito form in these compounds and slower kinetics at lower temperatures enabled experimental tracking of the nitro-to-*endo*-nitrito isomerization pathway.

We report the first experimental evidence of the previously predicted nitro-to-nitrito isomerization mechanism for square-planar nickel­(II) nitrite coordination compounds in the solid state. The formation of the *exo*-nitrito linkage isomer in the first stage of the photoreaction has been confirmed spectroscopically and structurally during extended irradiation with 530 nm LED, as well as via monitoring of both thermal and time relaxation processes. The experimental findings are supported by theoretical calculations performed for both compounds. The barriers for the nitro → *exo*-nitrito transformation (ca. 90.3 and 87.8 kJ·mol^–1^ for **Ni-4d** and **Ni-4d′**, respectively) significantly exceed the corresponding *exo*-nitrito → *endo*-nitrito reaction barriers (31.0 for **Ni-4d** and 28.3 kJ·mol^–1^ for **Ni-4d′**). Finally, the photoswitching reactivity of the studied systems is confirmed by determination of low-energy S_1_–S_0_ conical intersection points, easily accessible after the respective nitro-form excitation. Importantly, no direct photoreaction path between the nitro and *endo*-nitrito isomers was established, which indicates the sequential mechanism via the *exo*-nitrito intermediate.

In summary, the presented experimental evidence clearly shows that the nitrite group photoisomerization in square-planar Ni^II^ nitro complexes can be described as the nitro → *exo*-nitrito → *endo*-nitrito transformation in the solid state. This is in agreement with theoretical modeling and indicates that the mechanism *differs* from that proposed for the Ni nitro *octahedral* complexes. Nevertheless, there is still an open question whether an analogous mechanism of the nitrite group isomerization applies for other transition-metal square-planar nitro complexes. This will be further investigated.

## Supplementary Material













## Data Availability

High-volume raw and processed data deposited in the UW Research Data Repository under the following DOI: 10.58132/izx4cu.

## Notes

Comprehensive synthesis and compound characterization data, structure, crystal packing figures, supporting spectroscopic plots, cavity volume data, and additional computational data (PDF)
Ni-4d-series1 (CIF)
Ni-4d-series2 (CIF)
Ni-4d-prim-series3 (CIF)
Ni-4d-prim-series4 (CIF)
Ni-4d-prim-series5 (CIF)
