# Supplementary material for: Experimental Evidence for the Solid-State Nitrite-Ligand Photoisomerization Mechanism in Nickel(II) Square-Planar Complexes
Source: ACS Omega. 2025 Oct 26;10(43):51940–54. doi: 10.1021/acsomega.5c08267 (PMC12593162; doi:10.1021/acsomega.5c08267)
Supplement: Supplementary file 1 [file ao5c08267_si_001.pdf]

SUPPORTING INFORMATION

Experimental evidence for the solid-state nitrite-ligand photoisomerization mechanism in nickel(II) square planar complexes

Krystyna A. Deresz,[a,†] Artem Mikhailov,[b] Joanna Jankowska,[a,‡] Lorenzo Donà,[c] Bartolomeo Civalleri,[c] Adam Krówczyński,[a,†] Radosław Kamiński,[a,†] Dominik Schaniel,[b] Katarzyna N. Jarzembska [a,†*]

[a] University of Warsaw, Faculty of Chemistry, † Żwirki i Wigury 101, 02-089 Warsaw, Poland, ‡ Pasteura 1, 02-093 Warsaw, Poland

[b] Université de Lorraine, CNRS, CRM², 54500 Nancy, France

[c] Dipartimento di Chimica, Università di Torino, via Giuria 5, 10125 Torino, Italy

* Corresponding author: Katarzyna N. Jarzembska (katarzyna.jarzembska@uw.edu.pl)

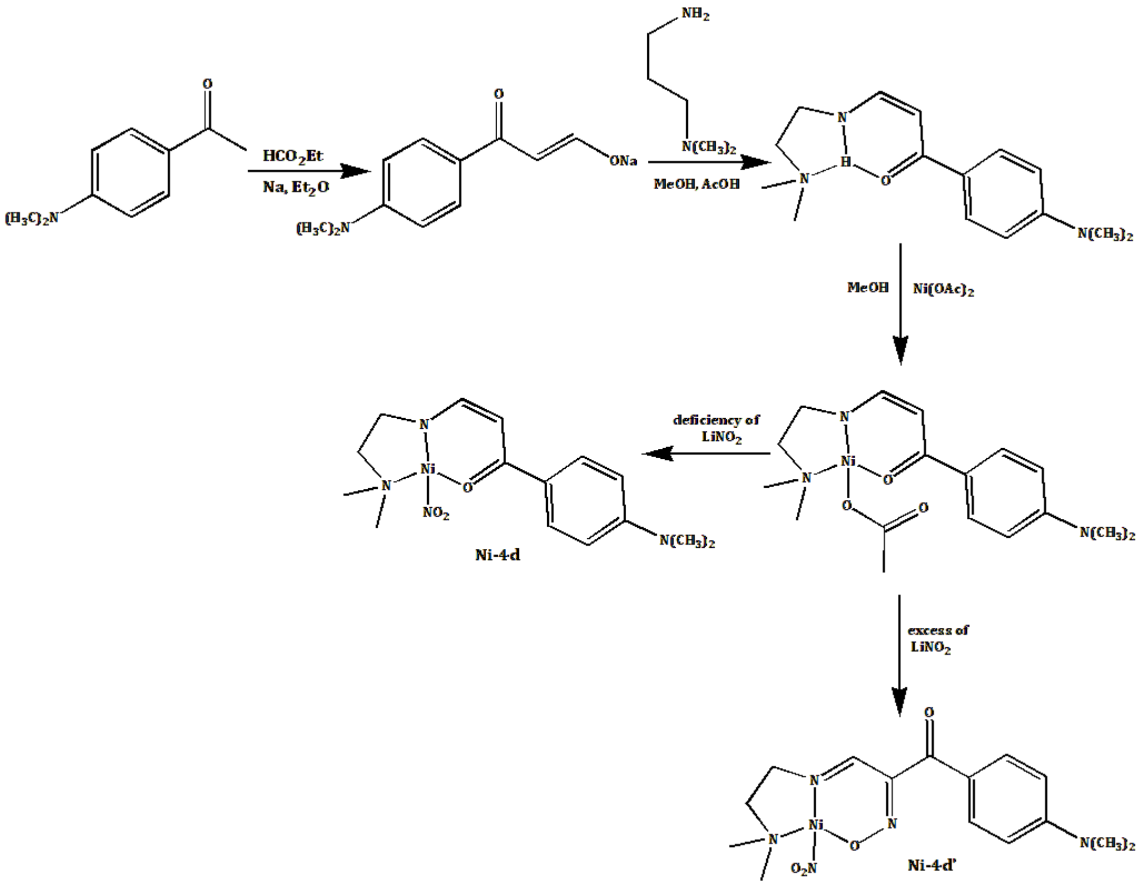

Scheme S1. Reaction scheme of the obtained compounds.

Table S1. Elemental analysis.

	Experimental			Calculated		
	C%	N%	H%	C%	N%	H%
**Ni-4d**	49.60	15.17	6.29	49.35	15.35	6.07
**Ni-4d'**	46.16	18.02	5.23	45.84	17.82	5.13

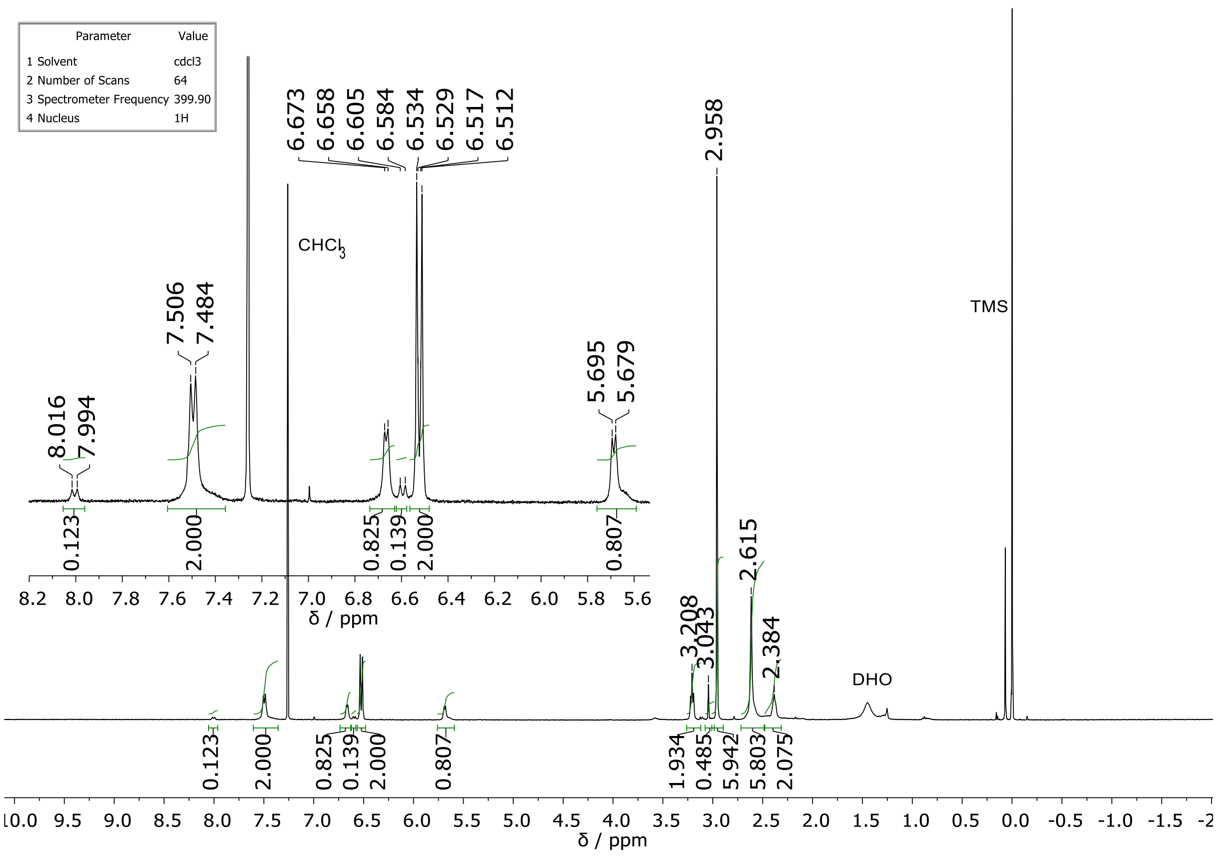

Figure S2. ^{1}H NMR spectrum of compound **Ni-4d** (400 MHz, $CDCl_3$).

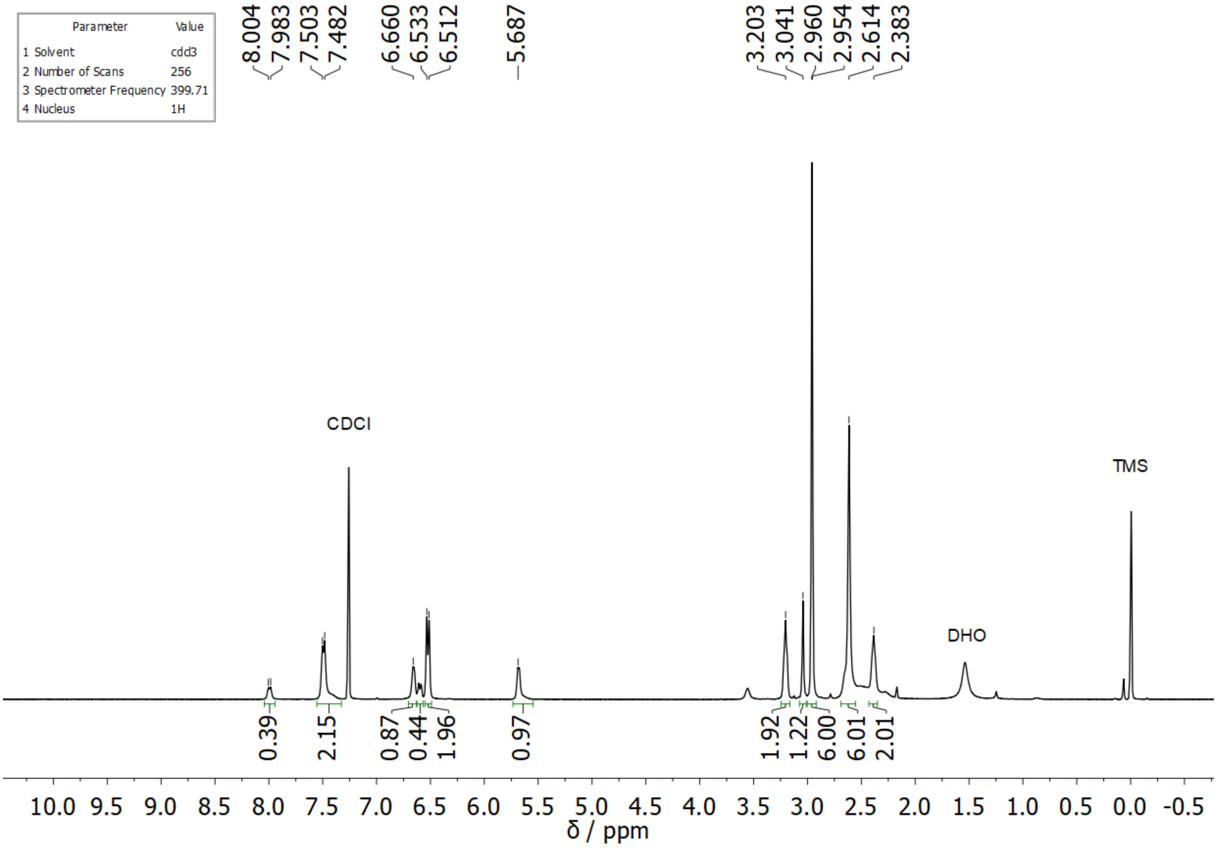

Figure S3. ^{1}H NMR spectrum of compound **Ni-4d'** (400 MHz, $CDCl_3$).

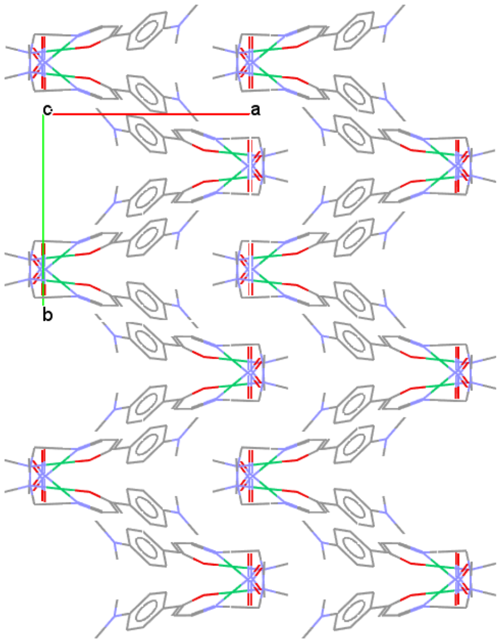

Figure S4. Herringbone motif in the **Ni-4d** crystal structure.

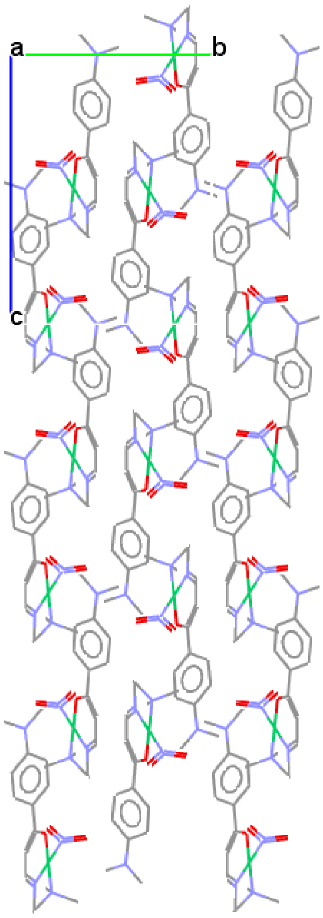

Figure S5. Ribbon-like motif in the **Ni-4d** crystal structure.

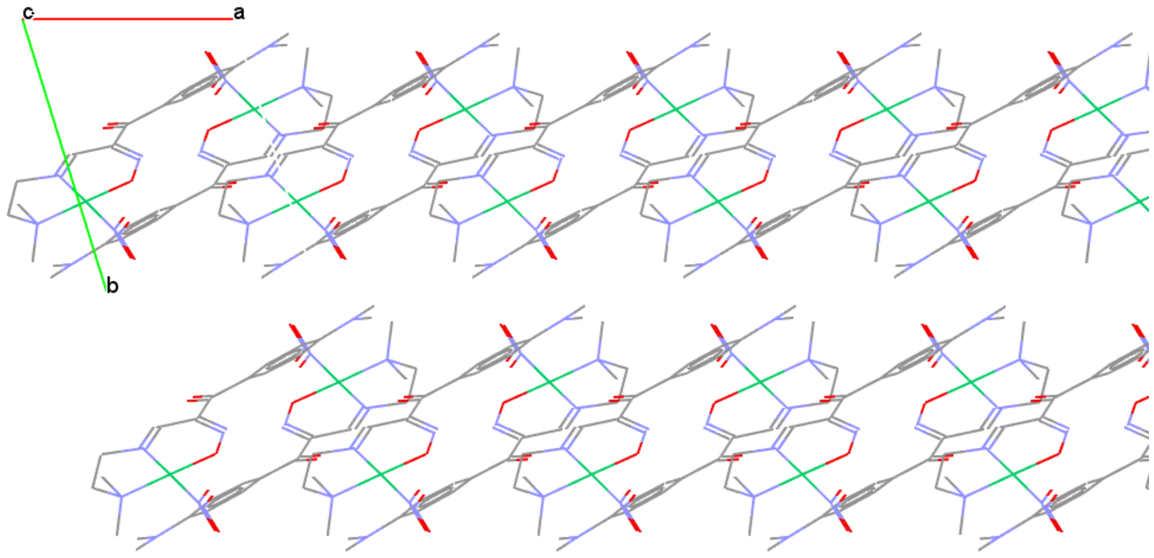

Figure S6. Dimeric plane motif in the **Ni-4d'** crystal structure.

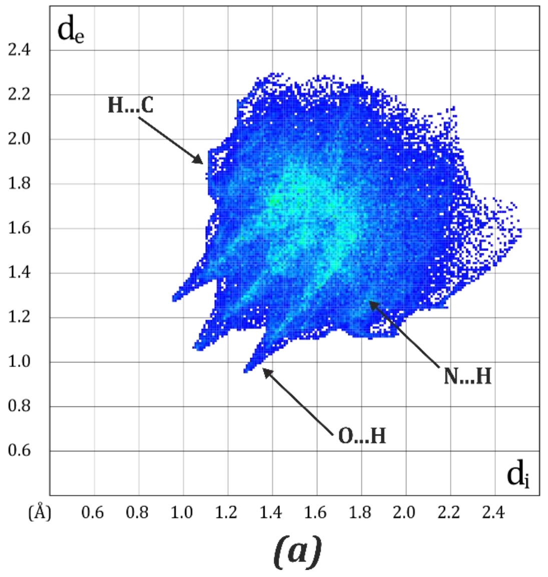

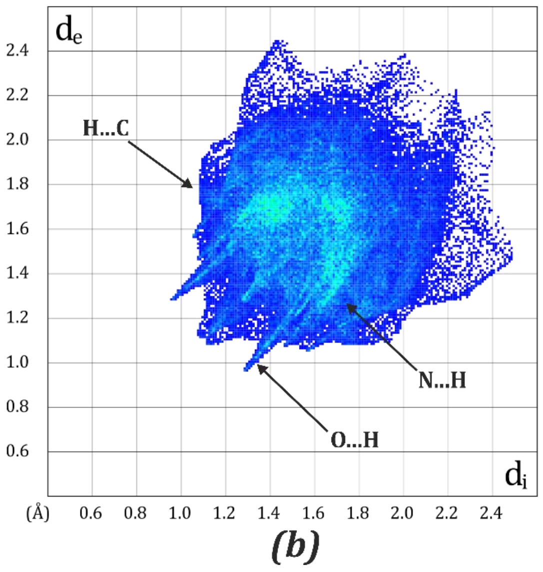

Figure S7. Fingerprint plots generated for two isomers of **Ni-4d'**: (*a*) nitro, (*b*) *endo*-nitrito.

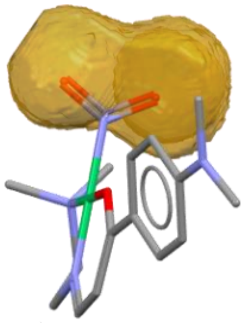

Figure S8. Reaction cavity of **Ni-4d**.

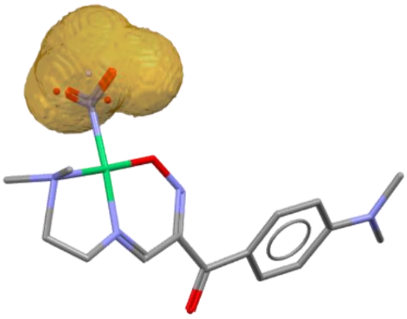

Figure S9. Reaction cavity of **Ni-4d'**.

Table S2. Approximate LED power densities used in IR experiments presented in the paper.

Central wavelength, λ_{mid} / nm	Power density, E_e / mW·cm^{-2}
660	125
590	34
530	71
470	217
405	188

Table S3. Key vibrational modes characteristic for the examined linkage isomers of the nitrite group with their theoretical counterparts for **Ni-4d**. All values given in cm^{-1}.

Isomer	*Vibration type*	*Theory*	*Experiment*
nitro-(η^1-NO_2)	ν_s	1384	1352
	ν_{as}	1486	1358
	ν_{as}	1507	1372
exo-nitrito-(η^1-ONO)	ν_s	1011	1068
	ν_s	1573	1436
endo-nitrito-(η^1-ONO)	ν_s	1087	1068
	ν_s	1108	1105
	ν_s	1121	1121
	ν_s	1490	1404
	ν_s	1495	1409

Table S4. Key vibrational modes characteristic for the examined linkage isomers of the nitrite group with their theoretical counterparts for **Ni-4d’**. All values given in cm^{-1}.

Isomer	*Vibration type*	*Theory*	*Experiment*
nitro-(η^1-NO_2)	ν_{as}	1378	1321
	ν_{as}	1451	1330
	ν_{as}	1468	1361
	ν_{as}	1481	1386
exo-nitrito-(η^1-ONO)	ν_{as}	958	1044
	ν_{as}	987	1065
	ν_{as}	1589	1460
endo-nitrito-(η^1-ONO)	ν_s	1049	1065
	ν_s	1080	1085
	ν_s	1092	1104
	ν_s	1096	1128
	ν_s	1489	1410
	ν_s	1497	1426

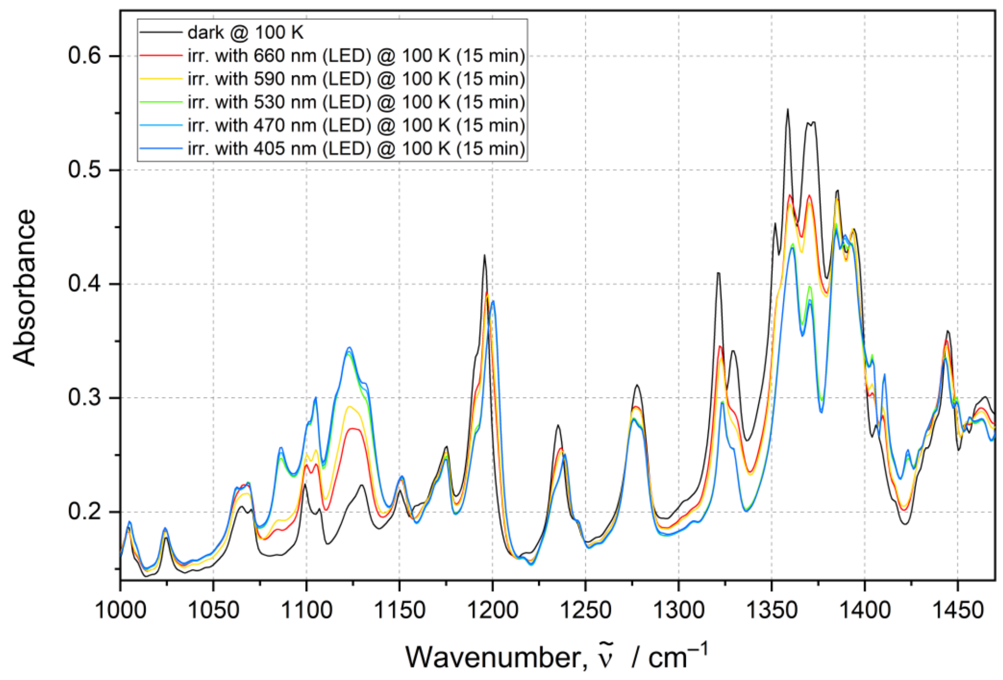

Figure S10. IR spectra collected for the **Ni-4d** sample at 100 K with before (black line) and after irradiation with: 660 nm LED (red line), 590 nm LED (yellow line), 530 nm LED (green line), 470 nm LED (light blue line), 405 nm LED (navy line).

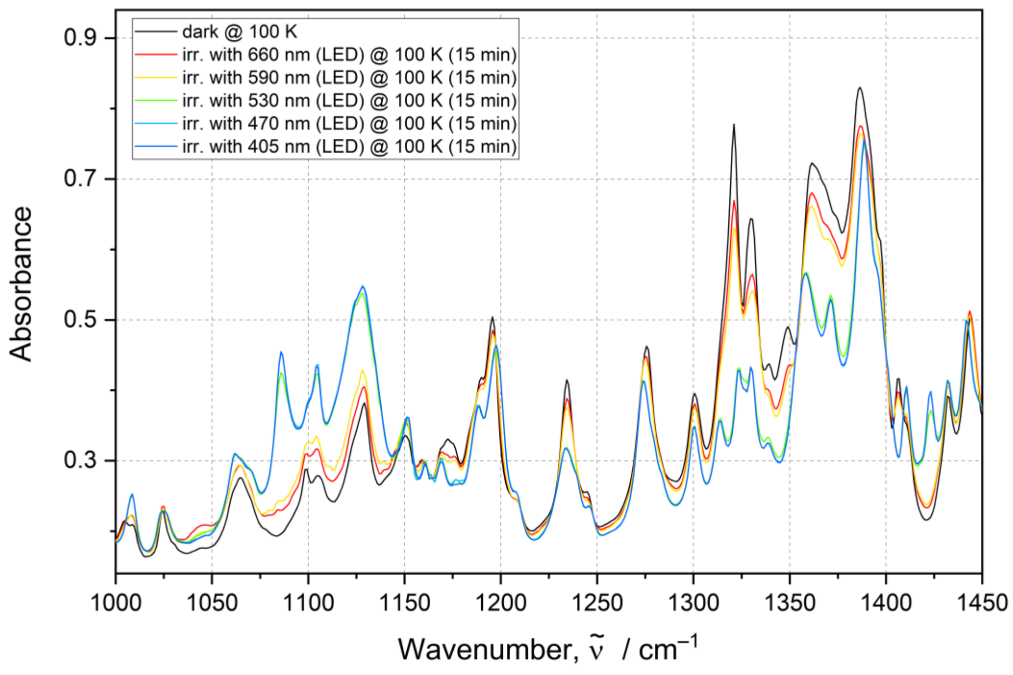

Figure S11. IR spectra collected for the **Ni-4d'** sample at 100 K with before (black line) and after irradiation with: 660 nm LED (red line), 590 nm LED (yellow line), 530 nm LED (green line), 470 nm LED (light blue line), 405 nm LED (navy line).

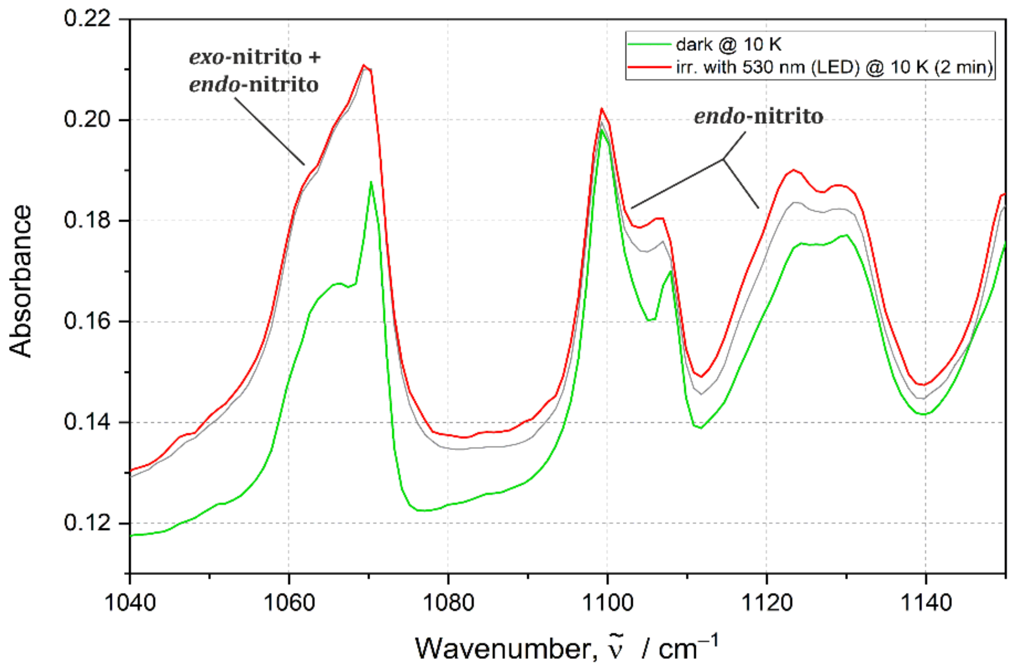

(a)

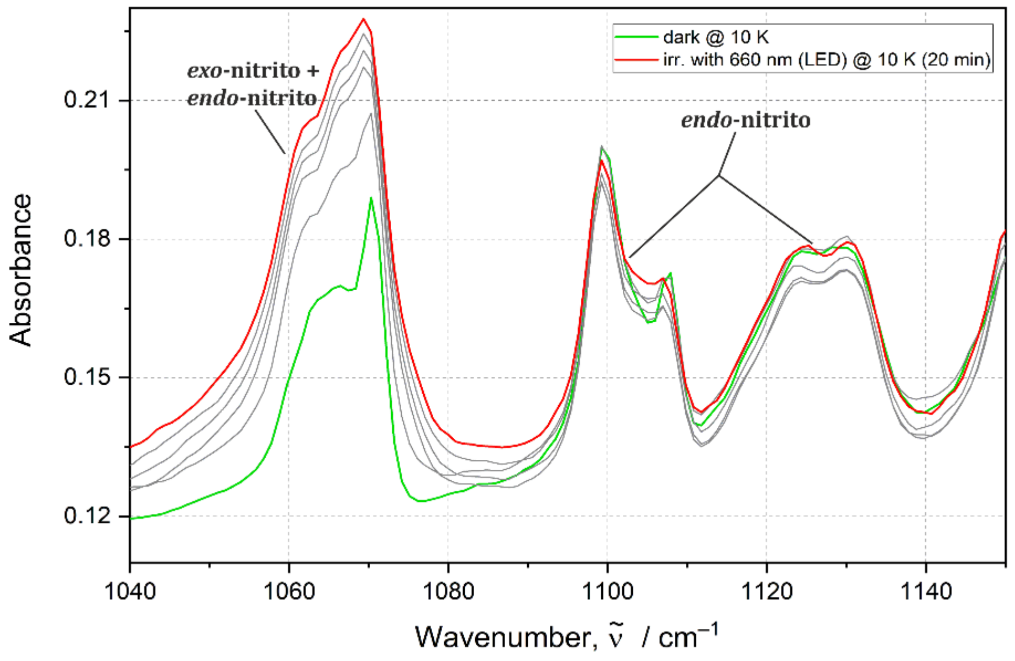

(b)

Figure S12. IR spectra collected before (green line) and after (red line) optimal irradiation time at 10 K for generating the *exo*-nitrito form in the **Ni-4d** sample: (*a*) with 530 nm LED sample; grey line correspond to subsequent irradiation point (1 min), and (*b*) with 660 nm LED; grey lines correspond to subsequent irradiation points (1–20 min).

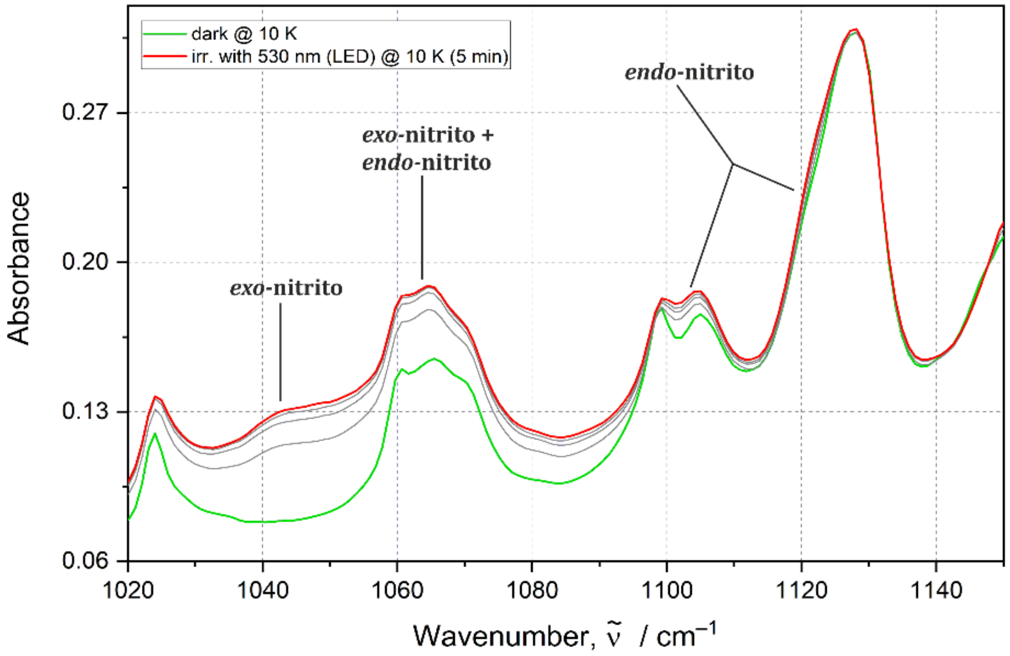

Figure S13. IR spectra collected before (green line) and after (red line) optimal irradiation time at 10 K for generating the *exo*-nitrito form in the **Ni-4d'** sample with 530 nm LED sample; grey line correspond to subsequent irradiation points (1–5 min).

Table S5. Summary of all collected data sets for the **Ni-4d** crystal structure. The codes correspond to the CIF files' names and their data blocks; T – temperature of the measurement, dark – data set without irradiation, irr – data set after irradiation (temperature of irradiation, LED wavelength, time of irradiation); number of experiment, and crystal ('xtal') No. at the end of the data set name.

Crystal No.	*Data collection code*	*CCDC code*	*Temp., T* / K	*Remarks*
**Ni-4d**, crystal 1 (**series 1**)				
1st	**Ni-4d-100K-dark-01-xtal1**	2433376	100	dark data collection
	**Ni-4d-100K-irr-530nm-1h-02-xtal1**	2433381	100	data collection after 1st 1 h of irradiation of the rotating crystal with the 530 nm LED
	**Ni-4d-100K-irr-530nm-2h-03-xtal1**	2433382	100	data collection after 2nd 1 h of irradiation of the rotating crystal with the 530 nm LED
	**Ni-4d-100K-irr-530nm-4h-04-xtal1** *1	2433380	100	data collection after additional 2 h of irradiation of the rotating crystal with the 530 nm LED
	**Ni-4d-140K-irr-530nm-4h-05-xtal1** *1	2433379	140	heating cycle
	**Ni-4d-160K-irr-530nm-4h-06-xtal1** *1	2433385	160	heating cycle
	**Ni-4d-180K-irr-530nm-4h-07-xtal1** *1	2433383	180	heating cycle
	**Ni-4d-200K-irr-530nm-4h-08-xtal1**	2433384	200	heating cycle
	**Ni-4d-220K-irr-530nm-4h-09-xtal1**	2433378	220	heating cycle
	**Ni-4d-240K-irr-530nm-4h-10-xtal1**	2433377	240	heating cycle
**Ni-4d**, crystal 2 (**series 2**)				
2nd	**Ni-4d-90K-dark-11-xtal2**	2433390	90	dark data collection
	**Ni-4d-90K-irr-660nm-30min-12-xtal2** *2	2433388	90	data collection after 30 min of irradiation of the rotating crystal with the 660 nm LED
	**Ni-4d-100K-irr-660nm-30min-13-xtal2** *2	2433387	100	heating cycle
	**Ni-4d-120K-irr-660nm-30min-14-xtal2** *2	2433389	120	heating cycle
	**Ni-4d-140K-irr-660nm-30min-15-xtal2** *2	2433386	140	heating cycle
	**Ni-4d-180K-irr-660nm-30min-16-xtal2** *2	2433391	180	heating cycle
	**Ni-4d-220K-irr-660nm-30min-17-xtal2**	2433392	220	heating cycle

Table S6. Summary of all collected data sets for the **Ni-4d'** crystal structure. The codes correspond to the CIF files' names and their data blocks; *T* – temperature of the measurement, dark – data set without irradiation, irr – data set after irradiation (temperature of irradiation, LED wavelength, time of irradiation). ; number of experiment, and crystal ('xtal') No. at the end of the data set name.

Crystal No.	*Data collection code*	*CCDC code*	*Temp., T* / K	*Remarks*
**Ni-4d'**, crystal 1 (**series 3**)				
1st	**Ni-4d-prim-100K-dark-18-xtal1**	2433395	100	dark data collection
	**Ni-4d-prim-100K-irr-530nm-2h-19-xtal1**	2433393	100	data collection after 1st 2 h of irradiation of the rotating crystal with the 530 nm LED
	**Ni-4d-prim-100K-irr530nm-4h-20-xtal1**	2433396	100	data collection after 2nd 2 h of irradiation of the rotating crystal with the 530 nm LED
	**Ni-4d-prim-150K-irr-530nm-4h-21-xtal1**	2433398	150	heating cycle
	**Ni-4d-prim-200K-irr-530nm-4h-22-xtal1**	2433397	200	heating cycle
	**Ni-4d-prim-220K-irr-530nm-4h-23-xtal1** [*3]	2433399	220	heating cycle
	**Ni-4d-prim-240K-irr-530nm-4h-24-xtal1** [*3]	2433400	240	heating cycle
	**Ni-4d-prim-260K-irr-530nm-4h-25-xtal1** [*3]	2433394	260	heating cycle
**Ni-4d'**, crystal 2 (**series 4**)				
2nd	**Ni-4d-prim-90K-dark-26-xtal2**	2433401	90	dark data collection
	**Ni-4d-prim-90K-irr-660nm-30min-27-xtal2**	2433403	90	data collection after 30 min of irradiation of the rotating crystal with the 660 nm LED
	**Ni-4d-prim-100K-irr-660nm-30min-28-xtal2**	2433406	100	heating cycle
	**Ni-4d-prim-120K-irr-660nm-30min-29-xtal2**	2433402	120	heating cycle
	**Ni-4d-prim-140K-irr-660nm-30min-30-xtal2**	2433714	140	heating cycle
	**Ni-4d-prim-180K-irr-660nm-30min-31-xtal2**	2433713	180	heating cycle
	**Ni-4d-prim-200K-irr-660nm-30min-32-xtal2**	2433715	200	heating cycle
	**Ni-4d-prim-220K-irr-660nm-30min-33-xtal2** [*4]	2433405	220	heating cycle
	**Ni-4d-prim-240K-irr-660nm-30min-34-xtal2** [*4]	2433404	240	heating cycle
**Ni-4d'**, crystal 3 (**series 5**)				
3rd	**Ni-4d-prim-280K-dark-35-xtal3** [*4]	2481491	280	dark data collection
	**Ni-4d-prim-250K-dark-36-xtal3** [*4]	2481492	250	dark data collection
	**Ni-4d-prim-220K-dark-37-xtal3** [*4]	2481488	220	dark data collection
	**Ni-4d-prim-190K-dark-38-xtal3** [*4]	2481489	190	dark data collection
	**Ni-4d-prim-160K-dark-39-xtal3** [*4]	2481493	160	dark data collection
	**Ni-4d-prim-130K-dark-40-xtal3** [*4]	2481487	130	dark data collection
	**Ni-4d-prim-100K-dark-41-xtal3** [*4]	2481490	100	dark data collection

Comment: To estimate more realistic e.s.d.s than the ones coming from the least-squares refinement we computed the average population over the selected set, as well as the population standard deviation: $\langle P \rangle_i$ and $(s_P)_i$, i – set number (set number is marked with ^{*i} in the preceding Tables 5S and 6S next to the data set name). It is the most reasonable to look at these uncertainties in relation to the respective average populations. These values were again averaged over all sets and yielded $\langle (s_P)_i / \langle P \rangle_i \rangle_{\text{all}} \approx 2.7\%$ for the chosen exemplary sets of data. Thus, one can state the e.s.d.s do not exceed 3%. This has been done just to obtain some idea of the uncertainties of the experimentally determined population values for the purpose of our study.

Table S7. Population of *endo*-nitrito form during X-ray multi-temperature measurements of **Ni-4d'** crystal structure.

Temperature, T / K	*endo*-nitrito population in %
280	27(1)
250	25(1)
220	25(1)
190	25(1)
160	27(1)
130	30(1)
100	31(1)

Table S8. Unit-cell parameters for the *CRYSTAL*-optimized crystal structures for each linkage isomer in **Ni-4d** and **Ni-4d'**.

	**Ni-4d**			**Ni-4d'**		
	nitro	*endo*-nitrito	*exo*-nitrito	nitro	*endo*-nitrito	*exo*-nitrito
a / Å	10.985	10.687	11.864	7.405	7.523	7.668
b / Å	10.856	10.687	9.570	9.848	10.349	10.307
c / Å	14.531	15.296	16.745	12.245	11.641	11.662
α / °	90	90	90	84.890	90.883	84.978
β / °	105.533	109.543	117.456	89.218	92.238	87.840
γ / °	90	90	90	71.018	70.155	69.510
V / Å^3	1669.446	1646.264	1687.032	840.906	851.829	860.055

Table S9. Reaction cavity volumes (V_{cav}) calculated for experimental and theoretical structures optimized with the *CRYSTAL* program.

	Reaction cavity volume, V_{cav} / Å^3			
Binding mode	**Ni-4d**		**Ni-4d'**	
	experiment	theory	experiment	theory
nitro	26.30	26.675	33.89	30.585
endo-nitrito	30.81	29.25	28.955	23.89
exo-nitrito	28.205	31.71	38.32	36.225

Table S10. Twist of the NO_2 ligand plane in respect to the rest of the molecule, computed as a torsion angle (τ) defined by the N2, Ni1, N1 and O1 atoms.

Binding mode	*Torsion angle*, τ / °	
	**Ni4d**	**Ni4d'**
nitro	85.80	81.45
endo-nitrito	88.72	92.93
exo-nitrito	73.93	111.40

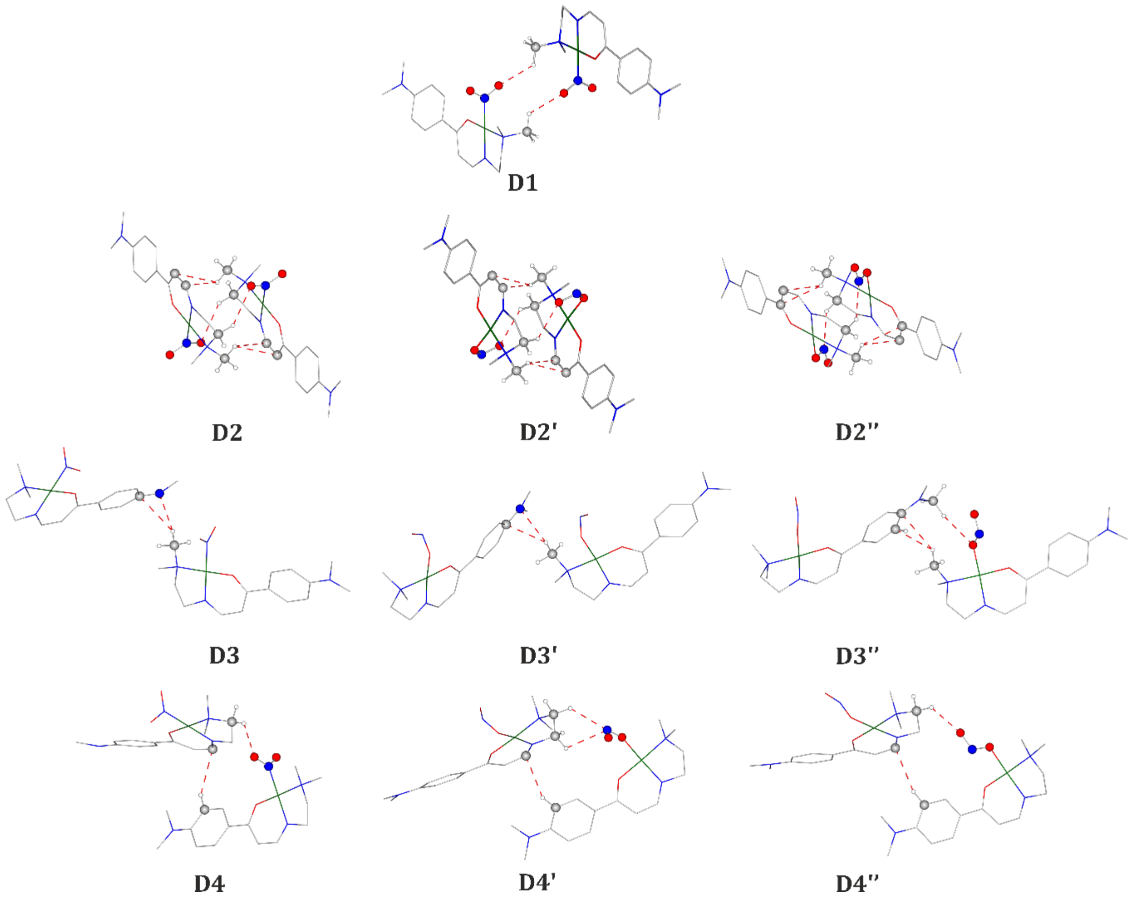

Figure S14. Main dimeric motifs in **Ni-4d** structure involving the nitrite ligand interactions encountered for each isomeric form. **D*x*** denotes a nitro motif, **D*x*'** – *endo*-nitrito motif and **D*x*''** – *exo*-nitrito motif (*x* – motif number).

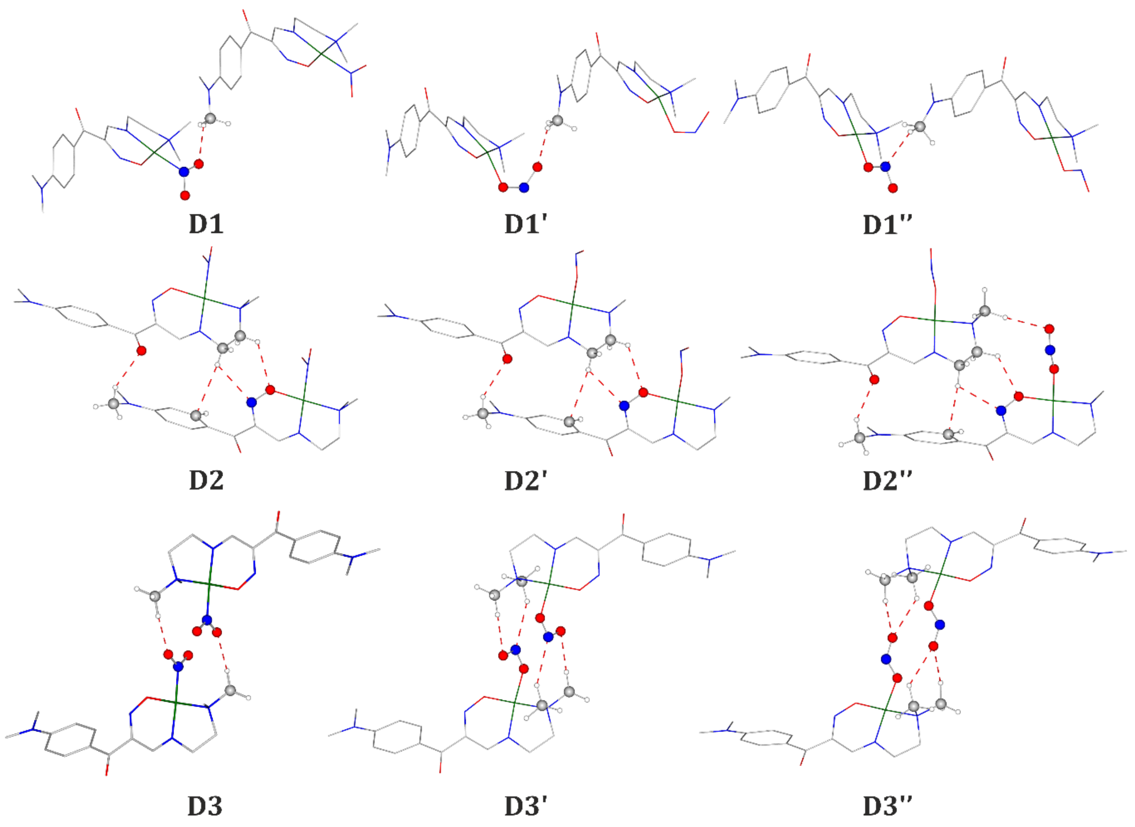

Figure S15. Main dimeric motifs in the **Ni-4d'** structure involving the nitrite ligand interactions encountered for each isomeric form. ***Dx*** denotes a nitro motif, ***Dx'*** – *endo*-nitrito motif and ***Dx''*** – *exo*-nitrito motif (*x* – motif number).

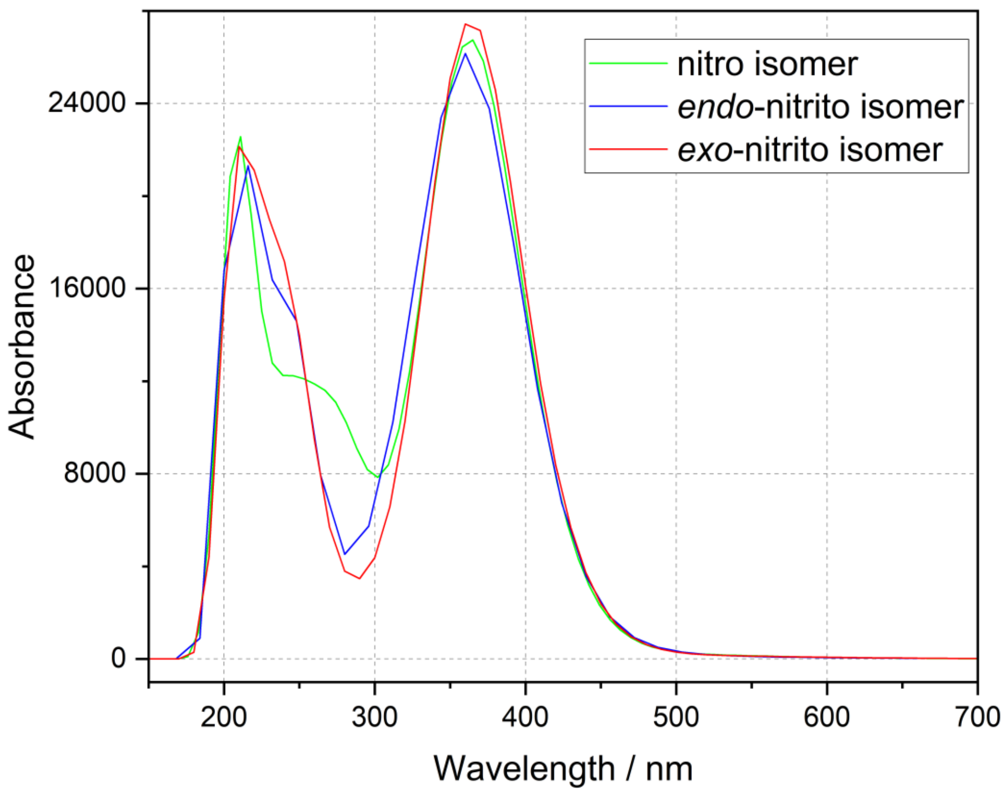

Figure S16. Theoretical UV-Vis spectra of three isomers of **Ni-4d**: nitro isomer (green), *endo*-nitrito isomer (blue), *exo*-nitrito isomer (red).

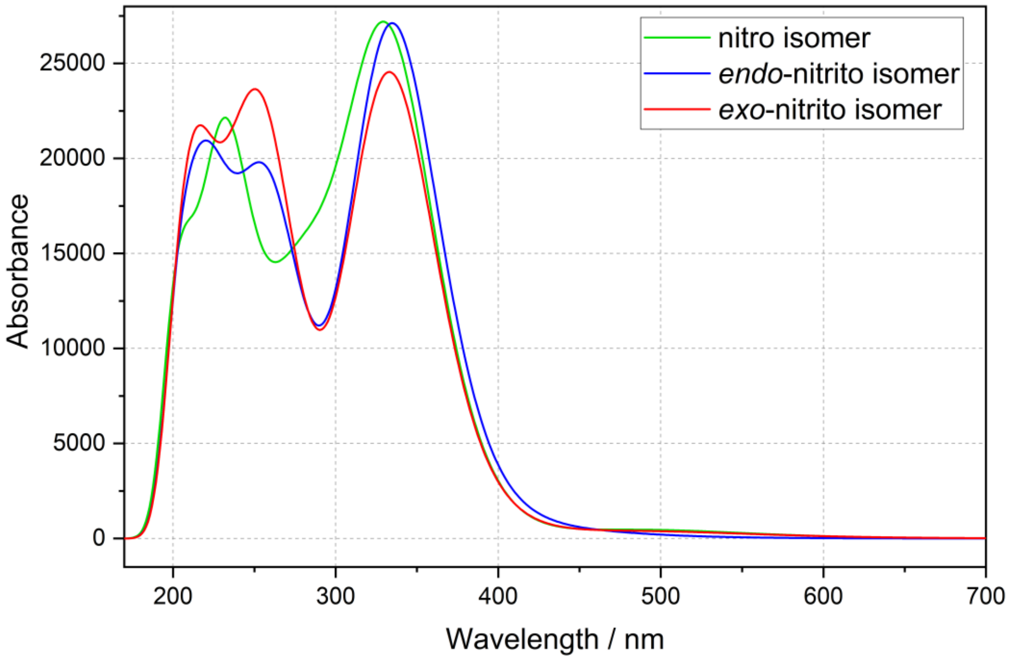

Figure S17. Theoretical UV-Vis spectra of three isomers of **Ni-4d'**: nitro isomer (green), *endo*-nitrito isomer (blue), *exo*-nitrito isomer (red).

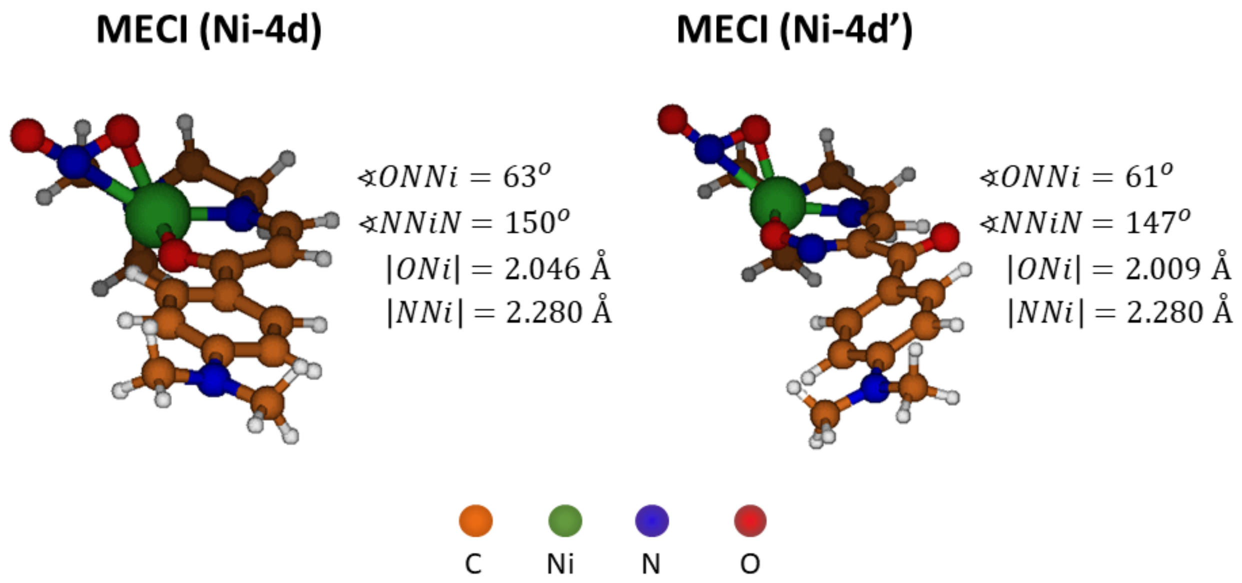

Figure S18. Approximate minimum-energy conical intersection (MECI) structures determined at the TDA-TDDFT level of theory with CIOpt/TURBOMOLE interfaced software suits. A B3LYP functional was employed with the three-body Grimme dispersion correction (D3), in combination with the 6-311++G** basis set applied for all atoms but the nickel, for which the 6-31G** basis set was used.
